# Interpretable lung-constrained RegNetY–ViT framework for pulmonary tuberculosis classification in chest X-rays with radiological feature-guided neuro-symbolic reasoning

**DOI:** 10.3389/fmed.2026.1908751

**Published:** 2026-09-10

**Authors:** Ibrahim Abdulrab Ahmed, Ebrahim Mohammed Senan, Awad Alyousef, M. Attique Khan, Elham Ali, Esam Mohammed Asem Othman, Suliman Mohamed Fati

**Affiliations:** 1 Department of Computer, Applied College, Najran University, Najran, Saudi Arabia; 2 Science and Engineering Research Center, Najran University, Najran, Saudi Arabia; 3 Department of Artificial Intelligence, Faculty of Computer Science and Information Technology, Al-Razi University, Sana’a, Yemen; 4 Department of Computer Science, College of Applied Sciences, Hajjah University, Hajjah, Yemen; 5 College of Computer and Information Sciences, Prince Sultan University, Riyadh, Saudi Arabia; 6 Department of AI Specialty, University College, Korea University, Seoul, Republic of Korea

**Keywords:** tuberculosis, chest X-ray, RegNetY–ViT hybrid model, Grad-CAM, lung-constrained segmentation, radiological biomarkers, neuro-symbolic fuzzy inference

## Abstract

**Introduction:**

Tuberculosis (TB) remains difficult to diagnose from chest X-rays due to overlapping radiological patterns with pneumonia, fibrotic scars, and other chronic lung abnormalities. Manual interpretation is highly dependent on expert experience and is often time-consuming, subjective, and prone to variability, especially in cases involving subtle or mixed lesion presentations.

**Methods:**

This study proposes a RegNetY–ViT hybrid framework for chest X-ray analysis using the TBX11K dataset. RegNetY captures fine-grained local spatial features such as cavitary margins, consolidation, and fibronodular patterns, while ViT models global contextual relationships across the lung fields. Interpretability is embedded within the pipeline through multi-lesion Grad-CAM, enabling the localization of multiple abnormal regions. These attention maps are further refined into lesion masks using lung-constrained segmentation with adaptive thresholding, active contour refinement, and overlap validation. Radiological biomarkers, including upper-lobe infiltrates, cavitary changes, heterogeneous consolidation, and fibrotic distortion, are extracted and fed into a neuro-symbolic fuzzy inference system to translate imaging features into rule-based diagnostic support.

**Results:**

The proposed hybrid model outperformed the standalone RegNetY and ViT models, particularly in detecting Active TB. It achieved an overall accuracy of 95.8%, macro-average sensitivity of 85.1%, macro-average specificity of 98.6%, and macro-average AUC of 87.2%. Interpretability analysis showed that Grad-CAM effectively localized disease-relevant lung regions, segmentation produced high consistency across lesion areas, and the neuro-symbolic layer generated clinically interpretable diagnostic explanations aligned with radiological biomarkers.

**Discussion:**

The proposed RegNetY–ViT hybrid framework improves TB classification performance while enhancing interpretability through lesion localization and neuro-symbolic reasoning, thereby supporting more transparent and clinically meaningful decision-making in chest X-ray analysis.

## 1 Introduction

The lung is a highly specialized respiratory organ where continuous physiological and immunological exchanges occur. It has an organized system of airways, alveoli, and vascular elements to support oxygenation and carbon dioxide homeostasis (1). In pulmonary tissue, *Mycobacterium tuberculosis* disrupts this balance, leading to chronic inflammatory and immune reactions. Over time, granuloma formation, caseous necrosis, and fibrotic remodeling progressively reduce ventilation efficiency (2). As the disease progresses, cavitation worsens the severity and spread. Chest radiography is widely used as a first-line imaging technique because of its availability and cost-effectiveness, especially in low- and middle-income countries (3). However, the radiologic patterns seen in TB can vary widely and are frequently confused with other pulmonary abnormalities (4); thus, they are difficult to interpret reliably, even for experienced clinicians.

Although significant achievements have been made in hybrid CNN, Transformer models, and explainable AI, one aspect of the methodology has yet to be fully addressed (5). Current models tend to decouple local features from global context modeling rather than embedding both in a common structure for interpretability. CNNs successfully capture fine-grained pulmonary abnormalities, and Vision Transformers encode global structural dependencies; however, their integration is mostly optimization-based and not through a clinical reasoning process.

If incorporated, interpretability is often limited to saliency maps based on Grad-CAM, which show the regions that were activated but do not check whether they correspond to clinically relevant biomarkers. These visual explanations still lack in medical decision support because they fail to provide consistent anatomical or pathological information (6). Furthermore, very little research has been performed and quantified in space to determine whether attention maps are correlated with clinically relevant tuberculosis biomarkers. Clinical reasoning is also largely symbolic; that is, there is a gap between visual explanation and medical logic.

The recent TB literature shows a rapid shift away from traditional CNN architectures toward hybrid and XAI architectures. Ou et al. (7) benchmarked several variants of the U-Net model, including Attention U-Net++ and PSP Attention U-Net, for semantic segmentation of TB lesions in chest X-ray images. When they adopted their ensemble framework, their MIoU and *F*1-scores were 0.70 and 0.81, respectively, thereby improving lesion delineation. However, owing to the small size of the training set, it may be difficult to extend the generalizability to more heterogeneous clinical populations. Sharma et al. (8) reported similar issues. They integrated U-Net lung segmentation with Xception classification and Grad-CAM visualization.

Performance optimization using feature engineering and hybrid fusion strategies has been the focus of several studies. Ali et al. (9) fused EfficientNetB0 and ResNet50 via fine-tuning, achieving an accuracy above 99% without interpretation. However, no external validation was performed. To enhance discriminative learning on small datasets, Kumar et al. (10) proposed an Orthogonal SoftMax Layer, whereas Ragavan et al. (11) introduced a hybrid optimization approach that combines the Sea Lion Optimization and Political Optimizer algorithms. These studies performed well in predictive terms but were less successful in terms of interpretation. The predictive performance is high, but the reasons for the predictions are not interpretable, thus hampering clinical trust and practical applications. Some studies were also based on internally curated, less diverse datasets, either in terms of demographics or pathologies, which might be less robust to changes in real-world distributions.

Subsequently, transformer-based and multimodal architectures have been developed to address the drawbacks of traditional CNN models. CNN-based systems showed good performance in capturing local pulmonary texture, whereas transformer-based models excelled at learning long-range context. To reduce computational redundancy, Lu et al. (12) proposed a CTBViT with Patch Reduction Blocks to preserve informative tokens. Ammar et al. (13) fused Vision Transformers and EfficientNet for TB recognition, and Kumar et al. (14) incorporated imaging and clinical information using cross-attention transformers. Kotei et al. (15) also used Data-efficient Image Transformer with ResNet-16 to obtain excellent diagnostic accuracy on TBX11K. In summary, all these studies highlight the methodological shift from performance-driven CNN-based architectures to context-aware hybrid systems. However, the validation of interpretability has not been sufficiently addressed.

Studies focused on interpretability have failed to fill this gap due to the lack of a thorough methodology. Rahman et al. (16), Maheswari et al. (17), and Nafisah et al. (18) employed Score-CAM, CAM, LIME, and visualization in explainable AI for TB classification. Nevertheless, most explanations remained at the level of visualization, lacking any quantifiable, clinically validated evidence. Visualization is insufficient without the possibility of quantification and clinical explanation. Statistical significance may not indicate the clinical significance of the findings. The most clinically relevant study was conducted by Chen et al. (19). Their analysis compared the work of AI-based models with that of pulmonologists working under abnormal circumstances in non-TB patients, when the diagnostic performance decreased.

Other studies have examined TB outside the field of imaging diagnostics. Qureshi et al. (20) combined SEIPR epidemiological modeling with NARX-based AI analysis to predict the development of multidrug-resistant TB. In contrast, Wang et al. (21) designed a genome-based deep learning analysis to predict drug resistance. Zheng et al. (22) employed machine learning algorithms to repurpose anti-TB drugs.

Previous research has shown a consistent tradeoff between accuracy and interpretability. In addition, ontology-based neuro-symbolic reasoning has not yet been used extensively to analyze TB images owing to its clinically grounded reasoning capabilities. To date, no literature presents an approach that uses hybrid CNN-ViT representation learning, quantitatively proven attention mechanisms, and ontology-based neuro-symbolic reasoning within a clinically interpretable tuberculosis imaging pipeline.

Previous studies on automated tuberculosis image analysis can be organized into four main computational directions. Segmentation-oriented methods have improved the localization and delineation of pulmonary abnormalities using U-Net-based architectures. However, these methods have mainly focused on identifying image regions rather than explaining how the extracted spatial information contributes to classification.

Feature-fusion and optimization-based approaches have reported strong classification performance through convolutional architectures, transfer learning, and specialized optimization strategies. Despite these improvements, many of these approaches provide limited information about the image patterns that influence the predicted class. Their outputs therefore remain difficult to trace beyond the final probability or class label.

Transformer-based and multimodal architectures have improved global-context representation and long-range dependency modeling. Nevertheless, local convolutional features and global transformer features are often combined without examining their relative contribution to the final representation. In addition, attention responses are commonly displayed as visual maps without objective evaluation of their spatial consistency.

Explainable-AI methods based on CAM, Score-CAM, Grad-CAM, and LIME have highlighted influential image regions. However, most studies rely primarily on visual inspection. Limited work has quantified the agreement between attention responses, pulmonary boundaries, lesion regions, and image-derived feature patterns.

Three connected computational challenges therefore remain insufficiently addressed. The first is the integration of local texture features and global spatial context through a transparent and trainable fusion mechanism. The second is the conversion of attention responses into measurable spatial regions rather than treating them only as visualization outputs. The third is the transformation of image-derived feature responses into structured and traceable rule-based evidence.

These unresolved challenges directly motivated the design of the proposed computational framework. The framework combines local and global representation learning, learnable feature fusion, multi-region attention analysis, lung-constrained lesion processing, quantitative spatial evaluation, feature-response extraction, and fuzzy symbolic inference within a unified image-classification pipeline.

The clinical application of AI-assisted diagnostic systems is not only about predicting accuracy but also ensuring that the decisions are transparent and medically interpretable.

The lack of clinical interpretation and validation of the model decisions is a significant limitation for accurate prediction alone. In this study, a novel, interpretable hybrid deep learning framework for chest X-ray classification, based on the RegNetY and ViT architectures, is proposed. The model combines local feature extraction via convolutional representations and global contextual modeling via transformer encoders to achieve complementary local–global representation learning.

Interpretable accuracy is an integral part of the design, whereas conventional systems aim solely for predictive accuracy. Diagnostically significant lung regions of interest (ROI) are identified using Grad-CAM biomarker attention maps. It is important to note that these visualizations are not used as independent explanations. A quantitative spatial validation module was added to assess the consistency between attention regions and clinically relevant tuberculosis biomarkers using IoU and spatial congruence. This enables quantitative evaluation of whether the model’s attention is focused on diagnostically significant pathological regions or on pseudo-image correlations.

Furthermore, ontology-guided neuro-symbolic reasoning was integrated to evaluate the predictions with structured radiological domain knowledge. Clinical rules based on medical ontologies assess whether detected imaging biomarkers align with known TB patterns, including apical involvement, nodular opacities, and cavitary lesions.

Principal methodological contributions:

The principal methodological contributions of this study are summarized as follows:

- A lightweight hybrid RegNetY–ViT architecture that combines fine-grained local feature extraction with global spatial-context modeling.
- A learnable weighted-fusion mechanism that integrates the convolutional and transformer representations within a shared feature space.
- A multi-lesion Grad-CAM procedure that identifies multiple spatially distinct regions contributing to the predicted class.
- A lung-constrained attention-processing strategy that limits the analysis to the estimated pulmonary fields and reduces responses outside the relevant image domain.
- An attention-guided lesion-processing method that converts continuous attention responses into refined binary regions using adaptive thresholding, active-contour refinement, and morphological operations.
- A quantitative explainability procedure that evaluates the spatial consistency of attention and lesion regions using overlap, pixel-level, and attention-coverage measures.
- An image-derived feature-response module that calculates, normalizes, and ranks selected radiographic patterns within the detected regions.
- A fuzzy neuro-symbolic inference procedure that integrates neural probabilities with structured rule-based evidence to produce a traceable computational output.

The remainder of this paper is organized as follows. Section 2 introduces the proposed RegNetY–ViT framework for analyzing TBX11K chest X-ray images, comprising preprocessing, lung masking, hybrid feature extraction, attention localization, lesion segmentation, biomarker analysis, and neuro-symbolic inference. In Section 3, we report that the proposed RegNetY–ViT model achieves strong performance on lung X-ray images, and we analyze the results of lung X-ray classification, Grad-CAM attention maps, lesion segmentation agreement, biomarker activation, and outputs for clinical inference. Section 4 compares the proposed model with existing CNN, transformer, segmentation, and explainable AI models, emphasizing its diagnostic and interpretative capabilities. Section 5 summarizes the study findings, main contributions, limitations, and future directions for clinical validation and explainable tuberculosis diagnosis.

## 2 Materials and methods

**Figure 1:**
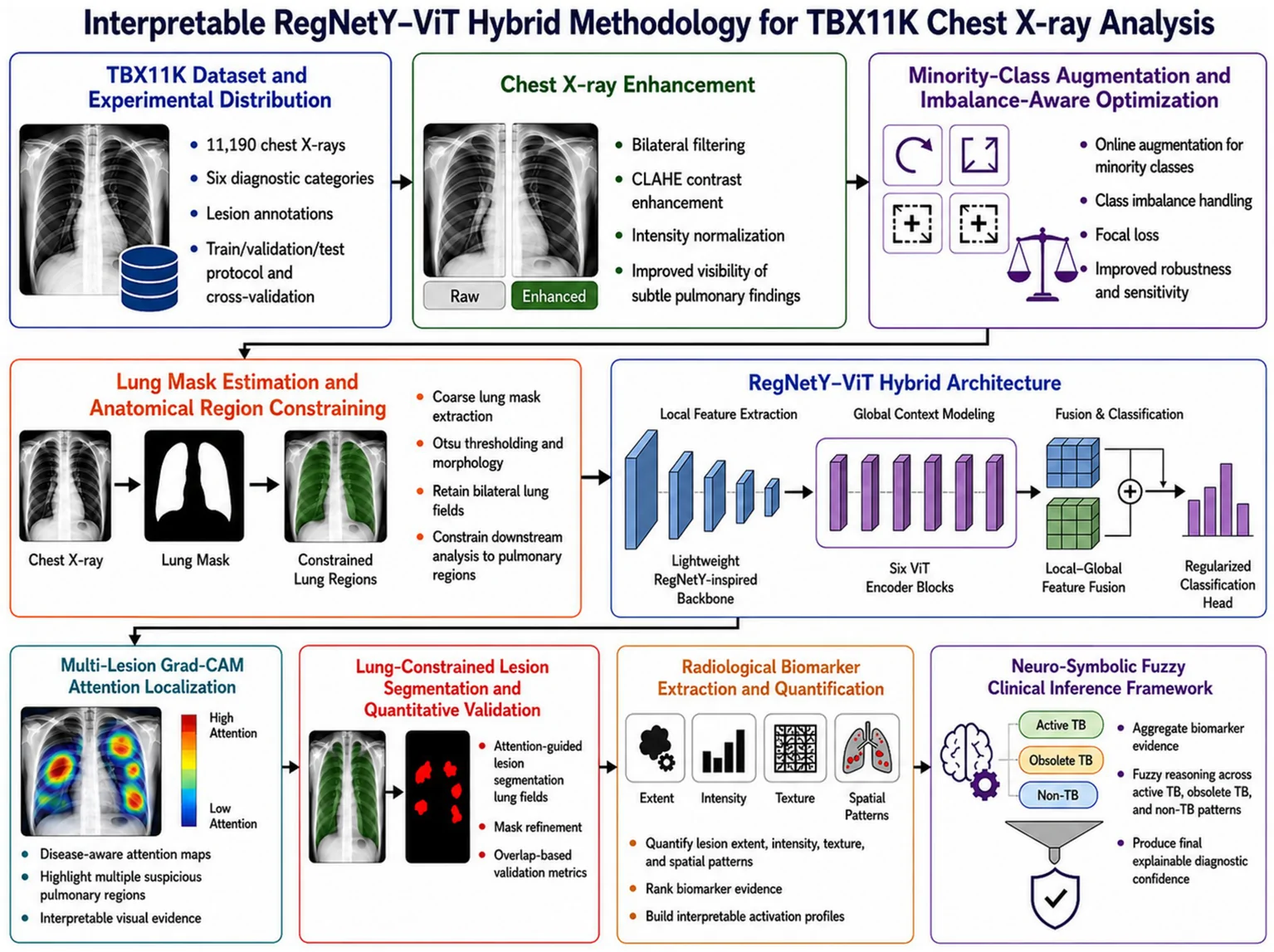
Overview of the proposed interpretable neuro-symbolic RegNetY–ViT hybrid framework for pulmonary tuberculosis analysis using TBX11K chest X-ray images.

As illustrated in Figure 1, the proposed and interpretable RegNetY–ViT framework follows a clear diagnostic pathway, starting with a TBX11K chest X-ray dataset and culminating in symbolic neuroclinical inference. First, images are resized, enhanced, and normalized to facilitate the analysis of subtle TB patterns, such as cavity borders, infiltrations, and fibrous tissue. Because the dataset contains rare TB subtypes, rotation, horizontal flipping, spatial shifting, zooming, and brightness adjustment were applied only to minority-class training images. Next, a lung mask restricts the analysis to pulmonary regions, preventing the model from being distracted by the ribs, back, or other irrelevant structures. The RegNetY branch captures local radiographic details, while the ViT branch examines broader spatial relationships across the lungs. These two forms of evidence are combined prior to classification. Grad-CAM then highlights suspicious lesion regions, which are further refined through lung-restricted segmentation and biomarker extraction. Finally, fuzzy symbolic neural rules translate visual findings into interpretable clinical evidence rather than silent prediction.

### 2.1 TBX11K dataset description and experimental data distribution

The release of TBX11K was attributed to the joint effort of scientists from Nankai University and InferVision. It comprises 11,190 chest X-ray images for tuberculosis-oriented artificial intelligence studies. The scale shifts the focus of the study. TBX11K contains five classes used in this study: Healthy, Sick_but_non_TB, Active TB, Latent TB, and Active & Latent TB. To minimize the processing time without sacrificing data quality, the images were rescaled to 512 × 512 pixels (23). Annotations include bounding boxes for lesions indicative of tuberculosis within the lung contours. Consequently, TBX11K transitions from a classification database to an imaging database that can be used for lesion localization, explainable AI, and hybrid diagnostic approaches (24).

The bounding boxes supplied with the dataset were defined by clinicians and indicate the approximate locations of tuberculosis-related pulmonary lesions. They represent rectangular spatial annotations rather than manually delineated pixel-level lesion contours. In the present study, these annotations were used as clinically informed spatial references for lesion localization and quantitative overlap assessment.

The TBX11K dataset was first divided at the patient level into an 80% development set and an independent 20% held-out test set. Table 1 reports the 80% development set and the independent 20% test set. Training and validation counts varied across the five cross-validation folds within the development set. Five-fold stratified patient-level cross-validation was then conducted only within the development set. In each fold, four subsets were used for model training, and one subset was used for validation. All radiographs belonging to the same patient were retained within a single fold and were not distributed between the training and validation subsets.

**Table 1:** Class distribution and patient-level partitioning of the TBX11K chest X-ray dataset.

Class name (disease)	Total images	Training images (65%)	Validation images (15%)	Test images (20%)
Healthy	5,000	3,250	750	1,000
Sick_but_non_TB	5,000	3,250	750	1,000
Active TB	924	601	138	185
Latent TB	212	138	32	42
Active and latent TB	54	35	8	11
**Total**	**11,190**	**7,274**	**1,678**	**2,238**

Bold values indicate aggregate or overall values.

The independent held-out test set was excluded from all cross-validation folds, data augmentation, hyperparameter optimization, early stopping, and model selection. It remained isolated throughout the model development process and was evaluated only once, after the final model configuration was selected. Accordingly, the reported test results represent a single evaluation on the fixed independent test set rather than an average across five test folds. This protocol reduced dependence on a single training–validation division while preserving patient-level independence between model development and final testing.

Five-fold cross-validation was used only for training, validation, and model selection within the development set; final evaluation was performed once on the fixed independent test set. Single-partition-dependent analysis can yield unreliable performance estimates, especially when applied to complex clinical problems such as medical image classification. In TBX11K, this issue is critical, as minor tuberculosis classes represent only a small number of examples, making their evaluation highly dependent on the choice of partitions. Cross-validation not only reveals potential overfitting but also helps to stabilize the performance estimates for each experiment.

The TBX11K dataset contains class imbalance, which is typical for medical imaging datasets and poses serious issues from both clinical and statistical perspectives. The major classes of healthy images and images showing signs of disease but not TB prevail in TBX11K, whereas rare subcategories of latent TB, and active–latent TB are extremely underrepresented. Imbalance is clinically important because the most challenging diagnostic cases are those of rare TB manifestations. Thus, imbalance-aware methods, including weight-based learning functions, focal loss, and data augmentation are necessary.

In addition to the diagnostic category labels, TBX11K provides bounding-box information indicating tuberculosis-related abnormal regions within chest radiographs. These bounding boxes were utilized in the present study as spatial reference information for lesion localization analysis, Grad-CAM validation, and quantitative evaluation of the explainability framework.

### 2.2 Enhancement of TBX11K chest X-ray images

The TBX11K Dataset contains raw chest X-ray images that exhibit substantial variation in illumination, image quality, lung contrast, and noise patterns across scanners. Imaging hardware, quantum mottle, compression artifacts, and variations in acquisition protocols can cause noise, whereas low local contrast can mask subtle markers of tuberculosis, such as micronodules, subtle infiltrates, fibrotic texture, and early cavitary boundaries (25). Image enhancement is more than visual preprocessing. It significantly affects the feature representation, model convergence, and stability of the explainable attention mechanism in deep learning analysis. To address these problems, a sequential preprocessing method using Bilateral Filtering (BF) and contrast-limited adaptive histogram equalization (CLAHE) was used.

First, a bilateral filter was applied to each chest radiograph to remove high-frequency noise while maintaining diagnostically significant anatomical edges (26). BF considers both radiometric similarity and spatial proximity between neighboring pixels when aggregating pixels. The filtered image $I_{\mathrm{BF}}(p)$ at pixel *p* is defined as in Equation 1:


$$I_{\mathrm{BF}}(p)=\frac{1}{W_{p}}\sum_{q\in S}I(q)\exp(-\frac{{|p-q|}^{2}}{2\sigma_{s}^{2}})\exp(-\frac{{|I(p)-I(q)|}^{2}}{2\sigma_{r}^{2}})$$ (1)

where I(p) is the intensity value of the target pixel, I(q) is the intensity value of the neighboring pixel *q* in spatial neighborhood *S*, $\sigma_{s}$ is a parameter that controls the spread of the neighborhood in space, $\sigma_{r}$ is a parameter that controls the radiometric similarity of the neighboring and target pixels, and $W_{p}$ is a normalization parameter that ensures that the intensities are consistent. The first exponential term penalizes pixels that are far away, whereas the second suppresses the average over large intensity transitions. Consequently, the pulmonary edges of the cavitary lesions, edges of the pleura, and fibrotic structures were preserved, and acquisition noise was minimized (27). To balance noise suppression and preserve subtle pulmonary abnormalities, $\sigma_{s}=1.5$ and $\sigma_{r}=25.0$ were chosen for the TBX11K images.

The denoised images on both sides were enhanced using CLAHE to improve lung contrast. CLAHE does not perform global histogram equalization of the image as traditional HEE does, but rather performs local histogram equalization in contextual regions, yielding better results in emphasizing subtle pulmonary textures without over-amplifying residual noise (28). CLAHE breaks the image into local contextual regions by partitioning it into an 8 × 8 tile grid and then applying a clip limit of 2.0 to prevent over-enhancement of these regions (29). The transformation function is expressed as follows (Equation 2):


$$T_{i,j}(v)=\frac{\sum_{k=0}^{v}H_{i,j}(k)-H_{\min}}{\sum_{k=0}^{L-1}H_{i,j}(k)-H_{\min}}\times (L-1)$$ (2)

where $T_{i,j}(v)$ is the intensity value of the original image that is mapped to the intensity value (*v*) in the tile (*i*, *j*); $H_{i,j}(k)$ is the histogram count of intensity level (*k*) in tile (*i*, *j*); $H_{\min}$ is the minimum histogram count after clipping; and *L* is the number of gray intensity levels. Because BF reduced noise, CLAHE could improve the contrast of TB-related structures without exaggerating irrelevant radiograph artifacts. After enhancement, intensity normalization was applied to further minimize the image intensity differences and improve the model convergence during training.

Therefore, the last enhancement framework is represented as follows (Equation 3):


$$I_{\text{Final}}=\text{CLAHE}(\text{Bilateral}(I_{\text{Input}}))$$ (3)

CLAHE, prior to denoising, accentuates noise patterns that are difficult to differentiate from real anatomical features. In contrast, the Bilateral → CLAHE sequence yielded cleaner pulmonary images, maintained diagnostically important edges, and produced more stable Grad-CAM attention localization of clinically relevant disease regions, but not high-frequency imaging artifacts.

### 2.3 Minority-class augmentation and imbalance-aware optimization

Similar to most medical imaging repositories, TBX11K is heavily imbalanced. As shown in Table 1, the majority of the images in the training sets are in the healthy and sick (but not TB) categories, with 3,250 images in each training set, while latent TB has 138 images, and combined active–latent TB has only 35 images. This imbalanced distribution results in a significant optimization bias during training (30).

To address class imbalance, data augmentation was applied only after completion of the patient-level partitioning procedure. Only radiographs assigned to the training set were augmented. No original or augmented image from the training patients was included in the validation or held-out test sets, and no augmentation was applied to either evaluation set (31). This order of operations preserved patient-level separation and prevented augmented versions of the same radiograph or different radiographs from the same patient from appearing across model development and evaluation subsets.

The training images from the minority classes underwent random rotation within 10°, horizontal flipping with anatomical plausibility in mind, width and height shifts within 10%, random zoom within 10%, and brightness adjustment within 15%. Stochastic augmentation was performed online during training, producing different transformations at each epoch without creating a fixed augmented dataset. The original validation and test images remained unchanged and were used exclusively for model selection and final independent evaluation, respectively (32). This helps avoid memorizing fixed transformed samples over epochs. Mathematically, the augmented image is represented by Equation 4.


$$I_{\mathrm{aug}}=T(I_{\mathrm{raw}};\theta_{r},\theta_{s},\theta_{z},\theta_{b})$$ (4)

where $I_{\mathrm{aug}}$ is the image of the chest radiograph after augmentation, $I_{\mathrm{raw}}$ is the training image, *T* (*·*) is the augmentation transformation function, $\theta_{r}$ is the rotation angle, $\theta_{s}$ is the spatial translation amount, $\theta_{z}$ is the zoom scale, and $\theta_{b}$ is the brightness adjustment amount.

This strategy can significantly increase the effective sample size of minority classes. This technique also minimizes overfitting by maximizing variation within the minority-class categories, which, in turn, reduces the variation between them (33).

However, it cannot completely resolve the imbalance problem. Despite augmentation, the majority of classes remained overrepresented, and the model might still overpredict health or sick-not-TB when it had a high degree of uncertainty. In the case of highly imbalanced data, the conventional cross-entropy loss tends to be driven by the majority classes (34). Therefore, Focal Loss was also used to optimize the model. Focal Loss differs from the common cross-entropy loss in that it decreases the loss for easy prediction examples and increases the loss for hard prediction examples, particularly those that belong to minority classes. Both approaches work at different stages of the learning process and hence complement each other. The Focal Loss formulation is expressed as follows (Equation 5):


$$\mathrm{FL}(p_{t})=-\alpha_{t}{\left(1-p_{t}\right)}^{\gamma }\log(p_{t})$$ (5)

where $p_{t}$ is the probability of the correct class, *γ* is a focus parameter to adjust the weight of the easy examples to be down-weighted, $\alpha_{t}$ is a class-balancing weighting factor inversely proportional to the class proportion in the training set, ${\left(1-p_{t}\right)}^{\gamma }$ is a modulation factor to suppress the loss contribution when $p_{t}$ approaches 1, and $\log(p_{t})$ is a classification cross-entropy term.

The modulating factor ${\left(1-p_{t}\right)}^{\gamma }$ gradually decreases the contribution of well-classified majority samples in the optimization process in Equation 5. If a sample is easy to classify, for example, a healthy lung with a high level of certainty, then $p_{t}\approx 1$ , and the loss is negligible. This mechanism helps enhance sensitivity to challenging tuberculosis manifestations. This is especially relevant in the context of false negatives predicted in the less well-represented subclasses of tuberculosis. However, if a sample is hard or a minority, the loss does not change and retains its size because the modulating factor is still close to 1 and $p_{t}$ is low. The weighting factor αt was set to be inversely proportional to the class frequencies in the training set. The selected value of *γ* = 2.0 guaranteed stable suppression of easy majority-class samples and did not destabilize the network convergence. This value has been extensively used in medical image classification to capture imbalance. Empirically, the hyperparameters were chosen to trade off between minority-class sensitivity and optimization stability (35).

The method enhances robustness to heterogeneous acquisition conditions and variations in pulmonary appearance. These strategies will collectively lead to better generalization, less optimization bias when the imbalance, and a more clinically reliable explainable tuberculosis classification.

### 2.4 Lung mask estimation and anatomical region constraining

In processed indiscriminately, chest radiographs can exhibit spurious correlations due to non-pulmonary structures such as the ribs, clavicles, mediastinum, heart, and background regions (36). The analysis was limited to the lung region by applying a coarse anatomical mask to the lungs, thereby reducing the effect of these structures (37). The goal of this stage was to create a lung-shaped search space, anatomically constrained, to be used in later stages for explainability, lesion localization, and biomarker analysis.

The normalized grayscale chest radiograph is defined as $I_{n}(x,y)$ , x∈[1,W] ; y∈[1,H] , where *W* and *H* are the width and height of the image in pixels, respectively. L(x,y) is a binary image defined as in Equation 6:


$$L(x,y)=\{\begin{array}{cc} 1, & (x,y)\in \Omega_{\text{lung}}\\ 0, & \text{otherwise} \end{array}$$ (6)

where $\Omega_{\text{lung}}$ is the estimated pulmonary field domain. The relationship between the mask and the domain, as in Equation 6a:


$$\Omega_{\text{lung}}=\{(x,y)|L(x,y)=1\}$$ (6a)

A hard anatomical constraint is modeled using Equation 6, which limits the analysis that follows to the estimated pulmonary field domain.

The first step in the estimation procedure was inverting the intensity domain. A normal chest radiograph shows dark regions, known as lung fields, surrounded by brighter regions representing denser tissue (32). The inverted image $I_{\mathrm{inv}}(x,y)=1-I_{n}(x,y)$ creates bright foreground regions in the lungs. High-frequency noise was suppressed while preserving the large-scale anatomical configuration using a Gaussian filter with a standard deviation of *σ* = 2 (as in Equation 7):


$$I_{\text{smooth}}(x,y)=(I_{\mathrm{inv}}*G_{\sigma })(x,y)$$ (7)

here, * denotes convolution, and $G_{\sigma }$ is the two-dimensional isotropic Gaussian kernel.

Otsu’s method uses the inter-class variance between foreground (candidate lung) and background (non-lung) pixels as a global threshold. The optimal threshold *T*_otsu_ that should be used is the threshold that satisfies, which is the same as Equation 8:


$$\sigma_{B}^{2}(T_{\text{otsu}})=\omega_{0}(T_{\text{otsu}})\omega_{1}(T_{\text{otsu}}){\left[\mu_{0}(T_{\text{otsu}})-\mu_{1}(T_{\text{otsu}})\right]}^{2}$$ (8)

where $\omega_{0}$ and $\omega_{1}$ are the class probabilities and $\mu_{0}$ and $\mu_{1}$ are their mean intensity values, respectively. To make the algorithm more robust when dealing with low-contrast radiographs and severe pathological presentations, the thresholding strategy used Otsu’s adaptive method with a lower bound, determined empirically (38), as in Equation 9:


$$T=\max(0.18,0.70\times T_{\text{otsu}})$$ (9)

In dark radiographs, a constant of 0.18 prevents the mask from collapsing completely. A factor of 0.70 was empirically determined to increase robustness with low-contrast radiographs and/or severe pathological presentations. $I_{\text{smooth}}(x,y)$ images were used to identify the approximate lung regions in the binary mask $M_{\mathrm{raw}}(x,y)$ using a smoothing filter, and the resulting mask was then compared with *T*.

The first binary mask usually had discontinuities, small artifacts, and broken regions. Therefore, a series of morphological operations was applied. A sequencing strategy was used, which closed with opening and then hole filling, to restore anatomical continuity first, followed by removing small-scale noise and preserving structures before connected component analysis (39). A disk-shaped (structuring element) with a radius of 8 pixels, and closed components in the lung that were close together. As in Equation 10:


$$M_{\text{close}}=(M_{\mathrm{raw}}⊕B_{8})⊖B_{8}$$ (10)

where ⊕ is dilation, ⊖ is erosion, and *B*_8_ is a disk-shaped structuring element with a radius of 8. Small protrusions and noise artifacts were isolated by opening with a radius-3 disk removed. Hole-filling operations were used to reconstruct anatomic continuity within the estimated lung regions.

Finally, a connected component analysis was applied to enforce the bilateral anatomy of the lung. Suppose that the set of connected components in the refined binary mask is $\left\{C_{1},C_{2},\ldots ,C_{K}\right\}$ , where the number of members of the connected component *C_i_* is denoted by $|C_{i}|$ . The two largest connected components were retained to approximate the bilateral pulmonary fields and to suppress small, isolated structures. The last lung mask is expressed as in Equation 11:


$$L(x,y)=\{\begin{array}{cc} 1, & (x,y)\in C_{\left(1\right)}\cup C_{\left(2\right)}\\ 0, & \text{otherwise} \end{array}$$ (11)

where $C_{\left(1\right)}$ and $C_{\left(2\right)}$ represent the largest and second-largest connected components, respectively. When the total number of lung pixels was less than 100, as in rare instances, a conservative approach was used to maintain downstream processing stability.

The estimated lung mask was then used to spatially constrain localization, segmentation, biomarker extraction, and explainability analyses, thereby confining the computations to the pulmonary regions. The anatomical constraint provides prior spatial knowledge that tuberculosis on radiographs should primarily appear in the pulmonary regions, not in non-pulmonary regions. By restricting the analysis to a subset of structures in the $\Omega_{\text{lung}}$ , the hypothesis space may be restricted, and the effect of non-pulmonary confounding structures may be reduced. The proposed masking procedure is computationally efficient, with a linear dependence between its complexity and image resolution, making it suitable for large-scale analysis of chest radiographs.

### 2.5 RegNetY–ViT hybrid architecture for pulmonary tuberculosis analysis

Perceptual skills are two complementary skills required for chest radiograph analysis. Local textures tell another story: the broken edge of a cavity, the delicate pattern of fibrotic scars, and the ground-glass mist of a developing infiltrate. The other teller is the spatial relationship evident at a global level: is an abnormality present in both apices, is the volume loss in the upper lobes related to fibrotic bands, and do consolidation and cavitation occur in the same anatomical zone? This duality cannot be fully captured by either CNNs or transformers (40). To address this, the proposed RegNetY–ViT hybrid architecture was designed to capture compact local features from RegNetY and long-range spatial dependencies using the ViT. To suppress non-pulmonary background structures and bias representation toward the estimated pulmonary field, the lung mask was applied prior to feature extraction.

#### 2.5.1 Lightweight RegNetY-inspired backbone for local feature extraction

Typical convolutional networks require significant computation. The RegNetY family is more structured: regularized design with efficient scaling. However, when the chest X-ray is a TBX11K (a large image), the discriminative information is not located in extreme spatial detail but rather in moderate-sized regions of the image, making the full RegNetY redundant.

Thus, a lightweight RegNetY-inspired backbone was designed. In typical RegNetY-like backbones, convolutional processing is often divided into several consecutive stages (41). A 3-stage lightweight variant was used in this study. The primitive spatial gradients of the grayscale chest radiograph were first captured using a 3 × 3 stem convolution (42). The first 3 × 3 stem maintained spatial gradient extraction along with the 1 × 1 compressing channels. The early stages consisted primarily of 1 × 1 convolutions for channel mixing and dimensionality reduction. The deepest stage had only 3 × 3 kernels, and larger receptive fields could combine local primitives into lesion patterns, such as cavitary margins, consolidation, and fibronodular opacities (43).

Allow for an improved chest radiograph, $I_{\text{final}}\in R^{H\times W\times 1}$ . The previous feature map of layer l is as in Equation 12:


$$F_{l}=\sigma (\mathrm{BN}(W_{l}*F_{l-1}+b_{l}))$$ (12)

The feature map at layer *l* is named $F_{l}$ , $W_{l}$ contains the convolutional kernels, the feature map is convoluted with each of the convolutional kernels, * denotes convolution, $b_{l}$ is the bias, BN is batch normalization, and σ(·) is the ReLU activation (44).

The cost of a convolutional layer grows as follows, as shown in Equation 13:


$$C_{\text{conv}}=O(K^{2}\cdot C_{\text{in}}\cdot C_{\text{out}}\cdot H\cdot W)$$ (13)

where *K* is the kernel size, $C_{\text{in}}$ is the input channel, and $C_{\text{out}}$ is the output channel. This cost is nine times lower if *K* is reduced to 1, which can be performed in the initial stages. Acceptable trade-off: TB lesions typically do not show up at single-pixel resolution.

The resulting output of the modified backbone is a spatial feature map $F_{\text{local}}\in R^{H^{\prime }\times W^{\prime }\times D}$ , where *D* = 256 is the feature dimension and *H'* and *W'* are the downsampled dimensions. This is a tensor that stores both the observed object and its location.

#### 2.5.2 Vision transformer for global context with six encoder blocks

CNNs learn local patterns well but not long-range dependencies. A cavity alone tells one story, and fibrotic changes on the other side tell another. To overcome this, the Vision Transformer enables every spatial location to attend to all other spatial locations.

The ViT branch was trained on feature maps instead of image patches. This option has two advantages. First, the CNN is already a rich local representation. Second, spatial dimensions are compressed, which reduces the number of tokens (45). The feature map $F_{\text{local}}$ was flattened and represented as a series of *N* = *H*' × *W*' vectors (46). The RegNetY branch scaled the feature map resolution down prior to transformer encoding while maintaining a lower number of tokens than the raw-image-patching approach. A projection of each vector was performed in an embedding space, as shown in Equation 14:


$$e_{p}=E_{\text{linear}}\cdot \mathrm{vec}{\left(F_{\text{local}}\right)}_{p}+e_{\mathrm{pos}}(p)$$ (14)

In this case, the embedding $e_{p}\in R^{d}$ for position *p*, the learnable projection matrix $E_{\text{linear}}\in R^{d\times D}$ , and the learnable positional encoding $e_{\mathrm{pos}}(p)$ are used. Positional encoding is essential (47). Otherwise, they would be used as an unordered set, which is unsuitable for the interpretation of a radiograph, where the position of the anatomical structures is significant in the diagnosis (48).

Multi-head self-attention is at the core of the transformer. Given query *Q*, key *K*, and value *V* matrices derived from the input embedding as in Equation 15 for a single head (41):


$$\text{Attention}(Q,K,V)=\text{softmax}(\frac{QK^{⊤}}{\sqrt{d_{k}}})V$$ (15)

where $d_{k}$ is the key vector dimension. Softmax generates attention weights for every position, enabling each token to capture information from the entire image. Multi-head attention is a parallelization of h attention operations as Equation 16:


$$\mathrm{MHA}(X)=\text{Concat}({\text{head}}_{1},\ldots ,{\text{head}}_{h})W^{O}$$ (16)

where ${\text{head}}_{i}=\text{Attention}(XW_{i}^{Q},XW_{i}^{K},XW_{i}^{V})$ . For TBX11K, the ViT branch employed six encoder blocks with an embedding dimension of 256 and eight attention heads. Each block comprised a pre-LN formulation of a multi-head attention (MHA) algorithm, followed by residual addition, and then a feed-forward network with another residual connection (as in Equations 17, 18):


$$X^{\prime }=X+\mathrm{MHA}(\text{LayerNorm}(X))$$ (17)

$$X_{\text{out}}=X^{\prime }+\mathrm{FFN}(\text{LayerNorm}(X^{\prime }))$$ (18)

The feed-forward network consisted of two linear layers with GELU activation and an expansion factor of 4. Six encoder blocks were stacked. *X* global ∈ *R N* × *d* is an output sequence with *N* tokens, where each token has global contextual information.

#### 2.5.3 Fusion of local and global representations

Currently, two streams flow side by side. The RegNetY branch offers spatially referenced local features. The ViT branch was used to produce global contextual embeddings. They need to be combined. Instead of uniformly concatenating or averaging the results, a learnable weighted fusion strategy was employed (49).

Let $F_{\text{local}}$ be the feature map extracted at the local level, and $X_{\text{global}}$ be the ViT output sequence. Separate projection functions, $\psi_{R}(\cdot )$ and $\psi_{V}(\cdot )$ , were used to project them into a common embedding space (46). The local feature tensor was first average-pooled across all of its structures, and the output of the ViT was mean-pooled across the tokens before being projected to $R^{256}$ . Both projection functions return vectors in $R^{256}$ , which must always have the same dimension before weighted fusion (50). Fusion was then defined as Equation 19:


$$F_{\text{fusion}}=\alpha \cdot \psi_{R}(F_{\text{local}})+(1-\alpha )\cdot \psi_{V}(X_{\text{global}})$$ (19)

The coefficient α∈[0,1] determines the contributions of the local and global features. Instead of being a fixed hyperparameter, *α* was made a learnable scalar parameter by applying a sigmoid function: α=σ(a) , where *a* is a trainable scalar parameter that is initially set to approximately α≈0.55 . This enables the model to learn the local–global trade-off (51).

The local tensor was global-average-pooled and the transformer tokens were mean-pooled; each pooled vector was then projected through a linear layer with 256 units. The output $F_{\text{fusion}}$ is a unified vector of dimension 256, combining local texture evidence with global spatial reasoning.

#### 2.5.4 Classification head with regularization

The fused representation must be converted into class probabilities. The classification pipeline included normalization, dropout, a multilayer perceptron, and softmax activation functions (52).

First, layer normalization ensured that the features had a consistent distribution, as shown in Equation 20:


$$f_{\mathrm{norm}}=\frac{F_{\text{fusion}}-\mu }{\sqrt{\sigma^{2}+\epsilon }}⊙\gamma +\beta$$ (20)

where μ and $\sigma^{2}$ are the mean and variance across features, *ϵ* prevents division by zero, and γ,β are learnable scale and shift parameters.

Dropout with rate p=0.3 was applied to prevent co-adaptation, as in Equation 21:


$$\tilde{f}=f_{\mathrm{norm}}⊙m,m∼\text{Bernoulli}(1-p)$$ (21)

A two-layer MLP with ReLU activation projected to the class logits, as in Equation 22:


$$z=W_{2}\cdot \text{ReLU}(W_{1}\cdot \tilde{f}+b_{1})+b_{2}$$ (22)

where $W_{1}\in R^{128\times 256}$ , $W_{2}\in R^{C\times 128}$ , and *C* = 5, corresponding to Healthy, Sick_but_non_TB, Active TB, Latent TB (radiographic obsolete pattern), and Active & Latent TB.

The final softmax layer converted logits to probabilities, as in Equation 23:


$$P(y=c\;I_{\text{final}})=\frac{\exp(z_{c})}{\sum_{j=1}^{C}\exp(z_{j})}$$ (23)

The predicted class was $\hat{y}=\arg\max_{c}P(y=c|I_{\text{final}}).$

#### 2.5.5 Training configuration

The details of the optimization setup for each of the three components of the suggested RegNetY–ViT model hybrid are listed in Table 2. Common among the branches are AdamW optimization, an initial learning rate of 1 × 10^−4^ with cosine annealing down to 1 × 10^−6^, a batch size of 16, and focal loss (*γ* = 2.0) to address class imbalance in TBX11K. The dropout values were different for RegNetY (0.2), ViT blocks (0.1 per block), and the classification layer (0.3). The difference in dropout values reflects varying regularization needs: the classification layer needs the most regularization, as it has the fewest parameters yet makes predictions. Stochastic depth (0.1) is used only for the six ViT encoder blocks to increase representation redundancy and prevent the collapse of compressed tokens. The weight decay was consistent at 1 × 10^−5^ for all branches. Training lasted for 100 epochs with early stopping (patience = 10), using the same hardware configuration (Intel Core i7-11700 CPU, GPU with 16 GB of VRAM).

**Table 2:** Optimization and regularization settings for the RegNetY-inspired branch, vision transformer branch, and classification head.

Parameter	RegNetY-inspired branch	ViT branch	Classification head
Optimizer	AdamW	AdamW	AdamW
Initial learning rate	1 × 10^−4^	1 × 10^−4^	1 × 10^−4^
Final cosine-annealing learning rate	1 × 10^−6^	1 × 10^−6^	1 × 10^−6^
Batch size	16	16	16
Maximum epochs	100	100	100
Early-stopping patience	10 epochs	10 epochs	10 epochs
Weight decay	1 × 10^−5^	1 × 10^−5^	1 × 10^−5^
Dropout	0.2	0.1 per encoder block	0.3
Stochastic depth	Not applied	0.1	Not applied
Loss function	Focal loss	Focal loss	Focal loss
Focal-loss focusing parameter (*γ*)	2	2	2
Learning-rate schedule	Cosine annealing	Cosine annealing	Cosine annealing
Checkpoint interval	Every 10 epochs	Every 10 epochs	Every 10 epochs
Model-selection criterion	Best validation performance	Best validation performance	Best validation performance

Training was performed on a machine with an Intel Core i7 Generation 11 processor, NVIDIA GPU 16 GB, and RAM 32 GB. The images used as input for the pipeline were 512 × 512 pixels. Model checkpoints were saved every 10 epochs, and the best-performing model was saved.

#### 2.5.6 Computational workflow and reproducibility

To improve methodological transparency and facilitate reproducibility, the complete computational pipeline is summarized in this subsection. The description focuses on the sequence and dependency of the processing stages without repeating the optimization settings and hardware configuration.

The pipeline begins with image standardization and enhancement. Each input image is resized to the required spatial resolution, filtered to reduce noise, and processed to improve local contrast. Intensity normalization is then applied to produce a consistent numerical range for the subsequent operations.

A pulmonary mask is estimated to restrict the computational analysis to the lung fields. The masking procedure combines intensity inversion, Gaussian smoothing, threshold estimation, morphological refinement, hole filling, and connected-component analysis. The valid pulmonary components define the spatial domain used by the feature-extraction and attention-processing stages.

The masked image is processed through two complementary representation branches. The RegNetY-inspired convolutional branch extracts local texture and structural patterns at multiple receptive-field levels. The Vision Transformer branch represents long-range spatial dependencies between distant image regions. Both outputs are projected into a common feature space and integrated using a learnable weighted-fusion mechanism.

The fused representation is passed to the classification head to estimate the probabilities of the five classes represented in the dataset. The learnable fusion weight allows the model to adjust the contribution of the local and global representations during optimization.

After classification, class-specific gradients are used to generate multi-lesion Grad-CAM responses. The resulting attention maps are constrained by the estimated lung mask and divided into separate search regions. This process allows multiple spatially distinct responses to be analyzed independently rather than being merged into a single diffuse activation map.

Each attention response is transformed into a candidate lesion region through a composite image score. The score combines attention intensity with selected intensity, edge, texture, and morphological responses. Adaptive thresholding produces an initial binary estimate, while active-contour refinement and morphological cleaning improve region continuity and remove small isolated components.

The refined regions are evaluated using quantitative spatial measures. These measures assess overlap, pixel-level detection behavior, and the proportion of high-attention pixels located within the corresponding regions. The evaluation therefore treats attention as a measurable computational output rather than relying only on visual inspection.

Image-derived feature responses are then calculated within each refined region. The responses are normalized and ranked according to their contribution. Fuzzy membership functions convert the continuous response values into structured symbolic evidence.

The final inference stage combines the neural class probabilities with the corresponding symbolic evidence. The framework returns the predicted class, probability distribution, attention responses, refined regions, spatial measures, ranked feature responses, and activated rules. This output structure supports traceable analysis of the computational sequence from the input image to the final model output.

Algorithm 1 summarizes the complete computational pipeline and the dependency between its principal processing stages.

**ALGORITHM 1:**
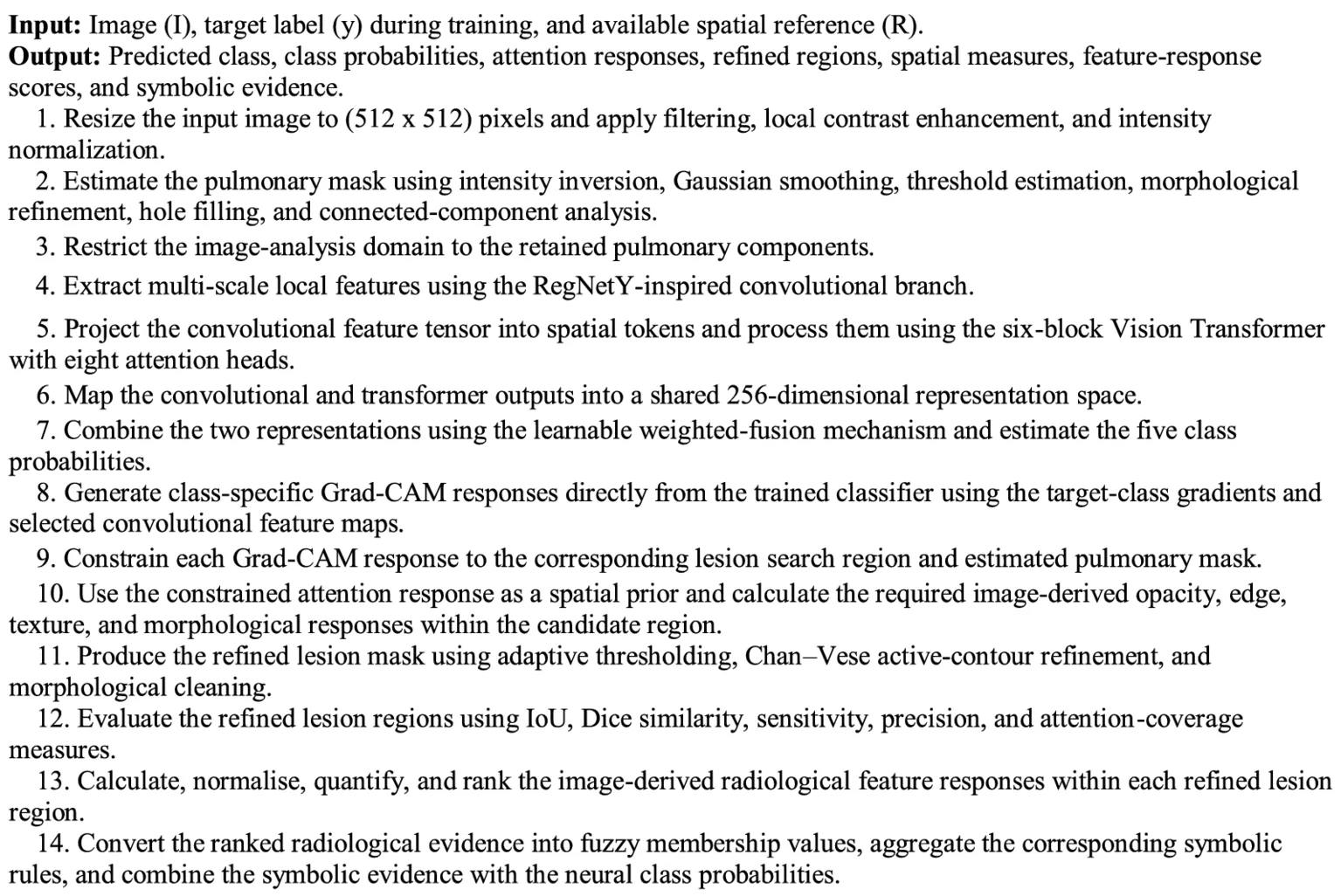
End-to-end computational pipeline of the proposed RegNetY–ViT framework.

### 2.6 Multi-lesion grad-CAM attention localization

After image enhancement, lung-field masking, and classification, the proposed RegNetY–ViT framework generated class-specific Grad-CAM responses to identify pulmonary regions contributing to the predicted class (53). The inputs to this stage were the enhanced chest radiograph, the estimated lung mask, the feature maps and target-class gradients obtained from the trained classifier, and the available lesion-specific search regions.

The Grad-CAM responses represented class-discriminative neural attention and were generated directly from the trained RegNetY–ViT model (54). No radiological biomarker maps were used as inputs at this stage. Clinician-defined bounding boxes were used to define lesion search regions; therefore, the resulting maps represent annotation-guided within-ROI localization rather than fully independent lesion detection. The resulting attention maps were used as spatial priors for subsequent lesion segmentation and did not determine which radiological biomarker was present (55).

The attention map does not simply serve as a decorative layer but rather as spatial evidence used in the final decision regarding radiographic abnormalities. The proposed method can simultaneously locate multiple regions of a pulmonary lesion, whereas conventional visualization can only locate a single region. Each lesion was processed separately in the search domain determined by lung size and then summarized in the visualization space (56).

A clinically guided search box, $B_{r}$ , is defined for each region *r* in which a lesion is located. The bounding region is not used as a ground-truth lesion mask, but only as an initialization seed to limit the internal search space. The effective pulmonary analysis domain is given by Equation 24, as follows:


$$\Omega_{r}=B_{r}\cap M_{\text{lung}},r=1,\ldots ,R,R\leq 3$$ (24)

The attention-processing procedure begins by spatially constraining the class-specific Grad-CAM response within the pulmonary search region. Gaussian smoothing is then applied to improve spatial continuity, followed by normalization, soft lung-constrained adjustment, gamma correction, and visualization. These operations are applied only to the neural attention response and do not alter the enhanced chest radiograph (57).

Let $F_{j}(x,y)$ denote channel *j* of the selected convolutional feature tensor, and let $z_{c}$ denote the logit corresponding to target class *c*. The importance weight of channel *j* for class *c* was calculated using the spatially averaged target-class gradient as in Equation 25:


$$\alpha_{j}^{c}=\frac{1}{Z}\sum_{x,y}\frac{\partial z_{c}}{\partial F_{j}(x,y)},$$ (25)

where *Z* denotes the number of spatial locations in the selected feature map.

The class-specific Grad-CAM response was then calculated as in Equation 26:


$$H_{c}^{\text{GradCAM}}(x,y)=\text{ReLU}(\sum_{j}\alpha_{j}^{c}F_{j}(x,y))$$ (26)

For each lesion-specific search region *r*, the raw attention response was constrained to the corresponding pulmonary domain, as in Equation 27:


$$H_{r}^{\mathrm{raw}}(x,y)=H_{c}^{\text{GradCAM}}(x,y)1_{\Omega_{r}}(x,y),$$ (27)

where $1_{\Omega_{r}}(x,y)$ is the indicator function of the intersection between the lesion search region and the estimated pulmonary mask. The resulting response was generated directly from the trained classifier and did not depend on radiological biomarker maps extracted during later processing stages.

Gaussian smoothing was applied to improve spatial continuity and flatten the fragmented activations, as shown in Equation 28:


$$H_{r}^{\text{smooth}}(x,y)=(H_{r}^{\mathrm{raw}}*G_{\sigma =7.0})(x,y)$$ (28)

here, $G_{\sigma =7.0}$ is an isotropic Gaussian kernel, and * denotes convolution. This operation is performed only on the lesion attention map to enhance spatial continuity, not to change the enhanced chest radiograph.

The smoothed attention map is then normalized inside the lesion-specific pulmonary domain, as described in Equation 29:


$${\hat{H}}_{r}(x,y)=\frac{H_{r}^{\text{smooth}}(x,y)-\min_{\Omega_{r}}(H_{r}^{\text{smooth}})}{\max_{\Omega_{r}}(H_{r}^{\text{smooth}})-\min_{\Omega_{r}}(H_{r}^{\text{smooth}})+\varepsilon }\cdot 1_{\Omega_{r}}(x,y)$$ (29)

where $1_{\Omega_{r}}(x,y)$ suppresses activations outside the lung-constrained region and ε is a numerical stability constant.

A soft lung-constrained adjustment was applied to retain low-to-moderate attention responses within the pulmonary search region. This operation reduced the risk of suppressing spatially weak but potentially relevant class-discriminative responses during normalization, as in Equation 30. It did not assign a radiological biomarker label to the activated region.


$$H_{r}^{\text{base}}(x,y)=0.82{\hat{H}}_{r}(x,y)+0.181_{\Omega_{r}}(x,y)$$ (30)

The effect of the nonlinear enhancement is computed using the gamma correction defined in Equation 31:


$$H_{r}^{\text{final}}(x,y)={\left(H_{r}^{\text{base}}(x,y)\right)}^{0.75}$$ (31)

The gamma parameter (0.75) is an empirically determined value to optimize low-to-moderate activation in regions of subtle lesions while preserving stability in highly activated regions (58).

Finally, disease-specific visualization is created by applying alpha blending, as shown in Equation 32:


$$V_{r}(x,y)=(1-\lambda )I(x,y)+\lambda C_{r}(H_{r}^{\text{final}}(x,y)),\lambda =0.70$$ (32)

where *λ* regulates the transparency of the original image and attention-guided overlay, and $C_{r}(\cdot )$ is a disease-dependent color mapping function.

The lesion-specific Grad-CAM maps provided spatial information about the pulmonary regions contributing to the predicted class. They were not treated as final lesion masks or as evidence of the presence of a specific radiological biomarker. Instead, they served as spatial priors for the lung-constrained lesion-segmentation procedure (59). The attention threshold was used to identify sensitive candidate regions, whereas the stricter segmentation threshold was applied before active-contour refinement and morphological cleaning.

### 2.7 Lung-constrained lesion segmentation and quantitative validation

Following class-specific Grad-CAM localization, the framework generated lung-constrained lesion regions. The attention responses indicated where the classifier concentrated its response, whereas the segmentation procedure estimated the spatial extent of the candidate abnormality (60).

The inputs to this stage were the enhanced chest radiograph, the binary lung mask, the class-specific Grad-CAM response, and the available lesion-specific search region. The attention map was used as a spatial prior rather than as a final lesion boundary. Image-derived opacity, edge, texture, and morphological responses were then calculated within the constrained pulmonary domain to generate the lesion-likelihood map (61).

Suppose $I_{e}(x,y)$ is an enhanced chest X-ray image at spatial coordinates (x, *y*), where *x* and *y* are the indices of the horizontal and vertical pixels, respectively, and Suppose that $M_{L}(x,y)$ is the binary lung mask, with value 1 indicating pixels retained for pulmonary analysis and 0 indicating excluded pixels such as image borders, mediastinal shadow, or background. For a single chest X-ray image, let k=1,…,K index the distinct lesions detected within that image, where K≤3 is the total number of lesions in the image, as in Equation 33.


$$\Omega_{k}=R_{k}\cap \{(x,y)|M_{L}(x,y)=1\}$$ (33)

where $\Omega_{k}$ restricts all subsequent segmentation processes exclusively to lung tissue within the clinically defined region of the lesion. This restriction minimizes the influence of extrapulmonary structures, image boundaries, and background distortions.

The composite lesion score combines five separate radiological cues in $\Omega_{k}$ . Let $O_{k}(x,y)$ be the normalized opacity response, defined as the local brightness measured with respect to the surrounding lung parenchyma, as follows: Let $G_{k}(x,y)$ be a measure of the strength of the edges at the lesion boundaries. Tuberculous lesions are more heterogeneous than healthy tissue; therefore, $T_{k}(x,y)$ is defined as the local texture variance computed from a 7 × 7 window using a standard deviation filter. $H_{k}^{\mathrm{top}}(x,y)$ is defined ined as the top-hat morphological response of an image taken with a disk structuring element *o*, of which 3, which accentuates small bright structures, such as nodules or consolidations. $H_{k}^{\mathrm{bot}}(x,y)$ is defined ined as the bottom-hat morphological response with the same structuring element, emphasizing dark or cavity-like structures in the image (62). They are all weighted and combined into a single lesion likelihood map $S_{k}(x,y)$ as described in Equation 34.

The composite lesion score combined the lung-constrained Grad-CAM response with five image-derived radiological cues. These cues represented opacity, edge strength, local texture variation, top-hat response, and bottom-hat response. For each lesion region *k*, the lesion-likelihood map was calculated as in Equation 34:


$$S_{k}(x,y)=\sum_{j=1}^{6}v_{j}F_{j}(x,y),(x,y)\in \Omega_{k},$$ (34)

$$\text{subject to}\;\sum_{j=1}^{6}v_{j}=1,v_{j}\geq 0.$$

In this equation, $F_{1}$ denotes the lung-constrained Grad-CAM response, whereas $F_{2}$ to $F_{6}$ denote the opacity response $O_{k},$ edge-strength response $G_{k},$ local texture response $T_{k},$ top-hat response $H_{k}^{\mathrm{top}},$ and bottom-hat response $H_{k}^{\mathrm{bot}},$ respectively. The coefficients $v_{j}$ are non-negative segmentation-cue weights satisfying $\sum_{j=1}^{6}v_{j}=1,v_{j}\geq 0.$ These coefficients are distinct from the radiological biomarker weights used during subsequent evidence quantification. The resulting lesion-likelihood score $S_{k}(x,y)$ was normalized to the range [0, 1] within the lung-constrained domain $\Omega_{k}$ before adaptive thresholding and active-contour refinement.

Adaptive thresholding determines the optimal cut-off for binarising the lesion likelihood map. Two complementary strategies are combined. First, Otsu’s method computes $\tau_{\text{Otsu}}$ to maximize the inter-class variance of $S_{k}$ within $\Omega_{k}$ . Second, percentile-based refinement computes $P_{62}(S_{k}\Omega_{k}),$ the 62nd percentile of lesion scores inside the same domain. This percentile was selected via empirical validation on a held-out subset to prevent over-segmentation in heterogeneous lesions. The final threshold $\tau_{k}$ takes the maximum of both values, as shown in Equation 35:


$$\tau_{k}=\max(\tau_{\text{Otsu}},P_{62}(S_{k}|\Omega_{k}))$$ (35)

The initial binary mask $M_{k}^{\left(0\right)}(x,y)$ is then obtained by applying this threshold and the domain constraint simultaneously:


$$M_{k}^{\left(0\right)}(x,y)=1_{\Omega_{k}}(x,y)\cdot 1[S_{k}(x,y)\geq \tau_{k}]$$ (36)

In this case, $1_{\Omega_{k}}(x,y)$ is the indicator function of the lung-constrained domain, and 1 [·] is the general indicator function, which takes the value 1 if its argument holds true, and is false.

The masks are often discontinuous or irregular when this threshold is used, and do not represent the contiguous nature of pulmonary lesions. To remedy this, a Chan–Vese active contour model was used to refine the initial masks. Suppose *C* is a developing closed contour, and $c_{1}$ and $c_{2}$ are the average pixel values inside and outside of *C*. The energy functional that is minimized during evolution is a compromise between contour length and intensity homogeneity, as follows from Equation 37.


$$\begin{array}{l} E(C)=\mu \cdot \text{Length}(C)+\lambda_{1}\int_{\text{inside}(C)}{|I_{e}-c_{1}|}^{2}\;dxdy+\lambda_{2}\\ \int_{\text{outside}(C)}{|I_{e}-c_{2}|}^{2}\;dxdy \end{array}$$ (37)

The boundary smoothness is controlled by the parameter μ , and the longer the contour, the more it is penalized. Parameters $\lambda_{1}$ and $\lambda_{2}$ balance the internal and external homogeneity terms, respectively, weighting the contour’s conformity to the intensity uniformity within and outside the image. These are implemented in the Chan-Vese active contour routine (63). The operator $\mathrm{AC}(I_{e},M_{k}^{\left(0\right)},n)$ refers to *n* = 35 iterations of this evolution, where at some point, the change in energy is below a tolerance *ε*. A high-quality ask is labeled $M_{k}^{*}$ .

The refined mask is cleaned in hological post-processing. Let C(·) be the morphological closing with a disk structuring element of radius, which fills small gaps and links nearby components. Let F(·) be an operator that fill seat are not filled in the lesion region. Let K(·) be the connected component removal operator that removes all connected components that have fewer than 5 pixels, and then preserves the two largest connected components. The preservation of two parts is clinically motivated because TB can present as a multifocal disease with a primary and satellite focus, and discarding the second largest part would be an error and would lead to a false-negative result. As in Equation 38, the lesion mask ${\hat{M}}_{k}$ is predicted at the end.


$${\hat{M}}_{k}=K(F(C(M_{k}^{*})))\cap \Omega_{k}$$ (38)

The last intersection with $\Omega_{k}$ reinforces the lung constraint after morphological operations.

To validate the mask, a quantitative comparison was performed between the mask and the reference. The reference lesion mask is denoted as $M_{k}^{\mathrm{ref}}$ , where *k* is the lesion of interest (bounding box). The intersection mask $I_{k}={\hat{M}}_{k}\cap M_{k}^{\mathrm{ref}}$ and union mask $U_{k}={\hat{M}}_{k}\cup M_{k}^{\mathrm{ref}}$ are defined as representing the number of pixels in the operator. Let ∣·∣ denote the pixel count operator. The Intersection over Union (IoU) is a measure of the ratio of overlap to the union of the two masks (Equation 39):


$${\mathrm{IoU}}_{k}=\frac{|I_{k}|}{|U_{k}|}$$ (39)

The Dice Similarity Coefficient provides an alternative overlap measure that is less sensitive to small changes in mask size (Equation 40):


$${\text{Dice}}_{k}=\frac{2|{\hat{M}}_{k}\cap M_{k}^{\mathrm{ref}}|}{|{\hat{M}}_{k}|+|M_{k}^{\mathrm{ref}}|}$$ (40)

Two other metrics measure the completeness of detection and purif localization (recall) measures the percentage of correct predictions for the reference lesion, and precision measures the percentage of the predicted lesion that actually appears, as shown in Equation 41.


$${\text{Sensitivity}}_{k}=\frac{|{\hat{M}}_{k}\cap M_{k}^{\mathrm{ref}}|}{|M_{k}^{\mathrm{ref}}|},{\text{Precision}}_{k}=\frac{|{\hat{M}}_{k}\cap M_{k}^{\mathrm{ref}}|}{|{\hat{M}}_{k}|}$$ (41)

For chest X-ray images containing multiple lesions within the same image—particularly those with both active tuberculosis and obsolete pulmonary tuberculosis findings—combined metrics are computed across all lesions in that image (64). Define the total predicted mask ${\hat{M}}_{\text{total}}=\overset{K}{\underset{k=1}{\cup }}{\hat{M}}_{k}$ and the total reference mask $M_{\text{total}}^{\mathrm{ref}}=\overset{K}{\underset{k=1}{\cup }}M_{k}^{\mathrm{ref}}$ where, ∪ denotes pixel-wise spatial union with overlap resolution via maximum pooling. Global IoU and Dice are then calculated using ${\hat{M}}_{\text{total}}$ and $M_{\text{total}}^{\mathrm{ref}}$ . This multi-lesion evaluation strategy preserves the clinical reality that tuberculosis may present as spatially distinct active and healed foci within the same radiograph.

### 2.8 Radiological biomarker extraction and quantification

After lesion segmentation and refinement, the framework quantified image-derived radiological patterns within each segmented pulmonary region. Segmentation defined the location and spatial extent of the candidate abnormality, whereas radiological feature quantification characterized the image patterns present within that region (65).

The inputs to this stage were the enhanced chest radiograph, the refined lesion mask, and the corresponding lung-constrained analysis domain (66). Image-derived responses were calculated for intensity, opacity, texture, edge structure, morphology, and anatomical location. The previously generated Grad-CAM response was retained only as an auxiliary attention feature and was not treated as a pre-existing biomarker map (67).

The resulting feature-response maps were normalized, quantified, and ranked to produce structured radiological biomarker evidence. These evidence scores were subsequently passed to the neuro-symbolic fuzzy inference procedure.

For each lesion region *k*, the raw biomarker response map is denoted by $F_{k,n}(x,y)$ . Here, n is the index of the biomarker channel. Each biomarker map was normalized within the pulmonary analysis domain using min-max normalization, as in Equation 42.

For each segmented lesion region *k*, the image-derived response associated with radiological pattern *n* was denoted by $F_{k,n}(x,y)$ . These response maps were calculated within the refined lesion region after segmentation. They were distinct from the class-specific Grad-CAM maps used earlier for spatial localization.


$$A_{k,n}(x,y)=\frac{F_{k,n}(x,y)-\min_{(u,v)\in \Omega_{k}}F_{k,n}(u,v)}{\max_{(u,v)\in \Omega_{k}}F_{k,n}(u,v)-\min_{(u,v)\in \Omega_{k}}F_{k,n}(u,v)+\epsilon }$$ (42)

Normalization restricts the biomarker response range in the pulmonary analysis domain to [0, 1]. This will minimize sensitivity to intensity variations across patients and ensure that a strong response to a less clinically important finding does not mask a weaker response to a more clinically important finding. If the maximum activation is zero, the normalized response will be zero, indicating that the radiological pattern has not been observed (68).

The mean activation of the biomarkers within the lesion domain was then determined, as Equation 43:


$$\mu_{k,n}=\frac{1}{|\Omega_{k}|}\sum_{(x,y)\in \Omega_{k}}A_{k,n}(x,y)$$ (43)

The corresponding peak activation is defined, as Equation 44:


$$\rho_{k,n}=\max_{(x,y)\in \Omega_{k}}A_{k,n}(x,y)$$ (44)

The mean activation, which reflects the extent of the spatial spread of the radiological pattern within the abnormal pulmonary region, is compared, and the peak activation is the strongest localized evidence. This is significant because TB can present as either a diffuse infiltrative pattern or focal cavitary or nodular lesions (69).

The biomarker relevance weights satisfy the following Equation 45:


$$\sum_{n=1}^{10}w_{n}=1,w_{n}\geq 0$$ (45)

The weights are the relative weights of the radiological biomarkers for lesion-level evidence. Pulmonary TB is more likely in those radiological patterns with a higher association with the disease, such as cavitary lesions, upper-lobe infiltrates, and fibronodular opacity, and less likely in those with a lower association, such as patchy consolidation (70).

The weighted biomarker support score is then calculated using the same method as in Equation 46:


$$S_{k,n}=w_{n}\cdot \rho_{k,n}$$ (46)

All biomarker channels are combined to form a composite “activation field” as in Equation 47, to generate a lesion-level evidence representation of the image.


$$H_{k}(x,y)=\sum_{n=1}^{10}w_{n}A_{k,n}(x,y)$$ (47)

This composite biomarker-evidence field was distinct from the earlier Grad-CAM attention map. The Grad-CAM response represented class-discriminative neural localization, whereas the composite biomarker field represented the weighted expression of image-derived radiological patterns within the segmented lesion. Regions with high values indicated strong support from one or more quantified radiological patterns and provided structured evidence for subsequent neuro-symbolic reasoning (71).

Finally, the biomarkers are ordered by decreasing levels of support, as shown in Equation 48:


$$R_{k}={\text{argsort}}_{\text{desc}}(S_{k,1},S_{k,2},\ldots ,S_{k,10})$$ (48)

A low-confidence threshold is applied to suppress responses from biomarkers that have only a small peak activation, thereby minimizing noise-dominated responses, while the highest-ranked biomarkers are retained for clinical reasoning. The ranked support scores were then converted into fuzzy rule activations, yielding interpretable statements in the following neuro-symbolic stage: strong cavitary evidence, moderate upper-lobe infiltration, or weak miliary involvement (72). Thus, this representation is a quantitative link between deep visual attention and explainable neuro-symbolic clinical inference.

### 2.9 Neuro-symbolic fuzzy clinical inference framework

This section receives the ranked biomarker vector $R_{k}$ and the support scores $S_{k,n}$ , rather than raw image features. Not raw image features, but rather a number of structured features were used. At this stage, no additional feature extraction is performed; only the module controls the previously extracted representations. The layer transforms quantitative evidence into a structured clinical inference: a predicted class, an evidence level, and the most influential explanatory rules (73).

Radiological biomarkers were divided into three clinical groups. The first set A represents active-TB markers (infiltrates, cavitation, consolidation, fibronodular opacity, lymphadenopathy, effusion, miliary pattern, volume loss, asymmetry, and small nodular changes). The second set O includes fibrotic scarring, calcified nodules, calcified granulomas, calcified hilar nodes, chronic fibrocalcific opacity, apical pleural thickening, residual fibrotic bands, old volume loss, and old architectural distortion, which are all considered obsolete TB markers (74). The third set N , refers to other markers (non-TB abnormalities) unrelated to TB, including cardiomegaly, pulmonary edema, hyperinflation, non-TB pleural effusion, and bronchial wall thickening. These sets are considered evidence sets and not singular discoveries.

Fuzzy logic is employed because the radiographic signs are not crisp binary events. Partially expressed apical opacity should not be classified as a strongly expressed cavitary opacity, even if there are signs of it. Therefore, each support score is converted into a fuzzy membership value using the sigmoid function (75). The membership value is not necessarily the strict probabilistic meaning as in Equation 49, but is a possibilistic interpretation of the contribution of soft evidence.


$$\mu (S)=\frac{1}{1+\exp[-\alpha (S-\beta )]}$$ (49)

where μ(S) is the fuzzy membership function, *α* determines the sharpness of the cliff from weak to strong membership, and β is the inflection point at which the membership value is 0.5. These parameters were treated as validation-dependent computational settings and were not interpreted as fixed clinical diagnostic cut-offs.

The weights $w_{n}$ are *a priori* defined clinical weights based on radiologic importance and are normalized within each evidence group during aggregation. The evidence for active TB in a lesion is calculated by weighted fuzzy aggregation over A, as shown in Equation 50:


$$E_{k}^{\text{active}}=\frac{\sum_{n\in A}w_{n}\mu (S_{k,n})}{\sum_{n\in A}w_{n}}$$ (50)

In this case, the lesion-level fuzzy evidence for active TB is $E_{k}^{\text{active}}$ . The normalization of all aggregated evidence scores is limited to between [0, 1]. The denominator allows the weights in the active TB group to be renormalized, enabling comparison of evidence scores across groups (76).

Similarly, as with the pulmonary TB evidence in Equation 50, obsolete evidence is treated as follows:


$$E_{k}^{\text{obsolete}}=\frac{\sum_{n\in O}w_{n}\mu (S_{k,n})}{\sum_{n\in O}w_{n}}$$ (51)

$E_{k}^{\text{obsolete}}$ obsolete are lesion-level evidence for healed or inactive post-tuberculous radiographic changes.

The first step was to estimate the evidence for non-TB abnormality by the non-TB biomarker group, as in Equation 52.


$$B_{k}=\frac{\sum_{n\in N}w_{n}\mu (S_{k,n})}{\sum_{n\in N}w_{n}}$$ (52)

where $B_{k}$ is the sum of the non-TB abnormality strengths. Finally, the last non-TB evidence is suppressed by a modulating mechanism, rather than by a strict probabilistic independence assumption as in Equation 53, when TB-compatible evidence is already strong enough to prevent assigning a non-TB diagnosis before TB is diagnosed.


$$E_{k}^{\text{nonTB}}=(1-E_{k}^{\text{active}})(1-E_{k}^{\text{obsolete}})B_{k}$$ (53)

where $E_{k}^{\text{nonTB}}$ is the lesion-level evidence of non-TB abnormalities. This multiplicative gating is a suppression mechanism that lowers non-TB evidence in the event of an active or old TB pattern.

The symbolic rule-based decision is then achieved by adjusting the thresholds, e.g., for Equation 54,


$$\begin{array}{l} {\text{Symbolic}}_{k}=\\ \left\{ \begin{array}{cc} \text{Active}\;\mathrm{TB}, & E_{k}^{\text{active}}\geq \theta_{\text{active}},E_{k}^{\text{obsolete}}<\theta_{\text{obsolete}}\\ \text{Latent}/\text{Obsolete}\;\mathrm{TB}, & E_{k}^{\text{obsolete}}\geq \theta_{\text{obsolete}},E_{k}^{\text{active}}<\theta_{\text{active}}\\ \text{Active}\&\text{Latent}\;\mathrm{TB}, & E_{k}^{\text{active}}\geq \theta_{\text{active}},E_{k}^{\text{obsolete}}\geq \theta_{\text{obsolete}}\\ \text{Sick}\;\mathrm{but}\;\text{non}-\mathrm{TB}, & E_{k}^{\text{nonTB}}\geq \theta_{\text{nonTB}},E_{k}^{\text{active}}<\theta_{\text{active}},E_{k}^{\text{obsolete}}<\theta_{\text{obsolete}}\\ \text{Healthy}, & \mathrm{no}\;\text{evidence group reaches}\;\mathrm{its}\;\text{decision threshold} \end{array}\right. \end{array}$$ (54)

Validation of adjustable decision thresholds: $\theta_{\text{active}}$ , $\theta_{\text{obsolete}}$ , and *θ* $\theta_{\text{nonTB}}$ ; symbolically adjustable decision at the lesion level: ${\text{Symbolic}}_{k}$ .

For multi-lesion images, evidence is aggregated at the image level using soft OR aggregation. This formulation describes the distributed pathology across multiple lesions, such that moderate findings in different regions add up, as in Equation 55.


$$E_{\text{image}}^{g}=1-\prod_{k=1}^{K}(1-E_{k}^{g}),g\in \{\text{active},\text{obsolete},\text{nonTB}\}$$ (55)

where $E_{\text{image}}^{g}$ is the evidence at the image level for group *g*.

Class-specific symbolic evidence is defined as the combination of the symbolic layer and the RegNetY–ViT classifier (77). These mappings ensure the logical consistency of the semantics of a class and the evidence for a group, as shown in Equation 56:


$$\begin{array}{l} E_{\text{image}}^{\left(q\right)}=\\ \left\{ \begin{array}{cc} E_{\text{image}}^{\text{active}}, & C_{q}=\text{Active}\;\mathrm{TB}\\ E_{\text{image}}^{\text{obsolete}}, & C_{q}=\text{Latent}/\text{Obsolete}\;\mathrm{TB}\\ \min(E_{\text{image}}^{\text{active}},E_{\text{image}}^{\text{obsolete}}), & C_{q}=\text{Active}\&\text{Latent}\;\mathrm{TB}\\ E_{\text{image}}^{\text{nonTB}}, & C_{q}=\text{Sick}\;\mathrm{but}\;\text{non}-\mathrm{TB}\\ 1-\max(E_{\text{image}}^{\text{active}},E_{\text{image}}^{\text{obsolete}},E_{\text{image}}^{\text{nonTB}}), & C_{q}=\text{Healthy} \end{array}\right. \end{array}$$ (56)

where the symbolic evidence assigned to class $C_{q}$ is $E_{\text{image}}^{\left(q\right)}$ .

The final neuro-symbolic confidence is calculated as a weighted combination (as Equation 57):


$$\Omega_{q}=\mathrm{\gamma P}(y=C_{q}|I_{\text{final}})+(1-\gamma )E_{\text{image}}^{\left(q\right)}$$ (57)

where $\Omega_{q}$ is the final neuro-symbolic confidence for class $C_{q}$ , and γ∈[0,1] is used to balance the neural probability with the fuzzy symbolic evidence.

The last prediction, as Equation 58:


$${\hat{y}}_{\mathrm{NS}}=\arg\max_{q}\Omega_{q}$$ (58)

Each activated biomarker rule is ranked. The larger the value of $\Pi_{k,n}$ , the more strongly it is activated, and the larger the number of *k* for which it is considered the top-k explanation, as in Equation 59:


$$\Pi_{k,n}=w_{n}\mu (S_{k,n})$$ (59)

where $\Pi_{k,n}$ is the explanatory rule score.

The top-ranked rules are listed in a human-readable format, for example, “Active tuberculosis is supported by moderate cavitary evidence and upper-lobe infiltrative evidence, but there is no dominant obsolete pattern. Thus, the layer did not regard chest radiographs as conclusive evidence of TB (78). It uses radiographic evidence to form a structured clinical reasoning process that remains uncertainty-aware and maintains the separation between active disease, obsolete post-tuberculous change, non-TB abnormality, and normal appearance.

### 2.10 Explainability validation framework

Explainability in chest radiograph analysis requires careful validation because visually convincing attention maps may still reflect nonclinical correlations or shortcut learning. In this study, Grad-CAM was used as an approximate localization tool rather than as causal evidence of the model’s reasoning. Therefore, the proposed framework evaluates explainability through a multi-stage verification process linking spatial attention, lesion localization, radiological biomarker evidence, and neuro-symbolic inference within the TBX11K dataset.

The validation framework receives four principal inputs from previous Sections: the final Grad-CAM attention maps $H_{r}^{\text{final}}$ , the predicted lesion masks ${\hat{M}}_{k}$ , the radiological biomarker scores $S_{k,n}$ , and the fuzzy inference outputs μ(S) .

The clinician-defined bounding boxes supplied with the TBX11K dataset were used as coarse spatial references rather than pixel-level lesion masks. These annotations indicate the approximate locations of pulmonary abnormalities but do not define exact lesion contours. This distinction is particularly relevant because tuberculosis-related abnormalities may have irregular or gradually merging boundaries.

For quantitative evaluation, each bounding box was represented as a binary rectangular reference region. The Grad-CAM responses and predicted lesion regions were compared with these references using spatial overlap and attention coverage measures. The resulting metrics assess localization consistency within the annotated regions. They do not measure agreement with exact expert-delineated pathological boundaries. To evaluate spatial agreement, the attention maps were first binarized using an activation threshold $\tau_{\mathrm{att}}=0.28$ , as in Equation 60:


$$H_{r}^{\mathrm{bin}}(x,y)=1[H_{r}^{\text{final}}(x,y)\geq \tau_{\mathrm{att}}]$$ (60)

The overlap between the activated attention region and the reference bounding box $B_{r}$ was then quantified using IoU, as in Equation 61.


$${\mathrm{IoU}}_{\mathrm{att},r}=\frac{|H_{r}^{\mathrm{bin}}\cap B_{r}|}{|H_{r}^{\mathrm{bin}}\cup B_{r}|}$$ (61)

Higher overlap values may suggest that the network attended to anatomically meaningful pulmonary abnormalities rather than unrelated structures, such as ribs, clavicles, or image borders. Nevertheless, overlap agreement alone was not taken as proof of clinically correct reasoning, as Grad-CAM provides only approximate spatial evidence. The attention coverage inside the predicted lesion region was further evaluated using Equation 62. Explanation stability was examined across the five patient-level cross-validation folds. Augmentation was restricted to the training portion of each fold, whereas explanation assessment was conducted on the corresponding non-augmented validation images. The independent held-out test set remained separate from this analysis until final evaluation.


$$\kappa_{\mathrm{att},k}=\frac{|\{(x,y):H_{r}^{\text{final}}(x,y)\geq \tau_{\mathrm{att}}\}\cap {\hat{M}}_{k}|}{|\{(x,y):H_{r}^{\text{final}}(x,y)\geq \tau_{\mathrm{att}}\}|}$$ (62)

where $\kappa_{\mathrm{att},k}$ measures the proportion of high-attention pixels located inside the refined lesion mask. Cases with $\kappa_{\mathrm{att},k}<0.5$ were considered spatially weak and flagged for interpretability review.

Similar disease categories were expected to activate relatively consistent pulmonary regions and produce compatible biomarker patterns across the folds. This analysis evaluates the explanation stability; however, it does not replace external validation of independent clinical datasets. Visual interpretability assessment was performed using a structured radiological plausibility review based on established TB-compatible radiographic manifestations, including cavitary opacities, apical infiltrative patterns, fibronodular changes, pleural thickening, and fibrocalcific abnormalities. Cavitary margins and fibrotic changes may gradually merge with the surrounding parenchymal opacity, making exact boundary interpretation difficult in some cases. Particular attention was given to activation outside the pulmonary regions because non-lung attention may indicate shortcut learning rather than clinically meaningful localization. Here, biomarkers refer to image-derived radiological patterns, rather than laboratory or molecular biomarkers. The biomarker evidence was evaluated jointly with Grad-CAM localization. For example, if the cavitary biomarker scores $S_{k,n}$ are strongly activated, the corresponding attention distribution is expected to concentrate near cavity-compatible pulmonary regions. Weak agreement between spatial attention and biomarker evidence suggests potentially unstable or indirect dependence on features. The neuro-symbolic verification stage examined whether the final prediction was logically consistent with the activated biomarker groups. Rule agreement was quantified using, as in Equation 63: Within the fuzzy framework, a rule such as “strong cavitary evidence with limited obsolete-pattern evidence” increases the symbolic support for active tuberculosis, whereas dominant fibrocalcific and chronic fibrotic evidence increases the support for obsolete tuberculosis.


$$\phi_{\text{rule}}=\frac{1}{N_{\text{test}}}\sum_{i=1}^{N_{\text{test}}}1[{\text{Rules}}^{\left(i\right)}\subseteq {\text{ClinicallyAcceptable}}^{\left(i\right)}]$$ (63)

These rules provide uncertainty-aware interpretability support rather than a deterministic diagnosis. The resulting framework establishes a traceable interpretability pathway that connects enhanced chest radiographs, Grad-CAM localization, lesion-aware segmentation, biomarker extraction, and neuro-symbolic reasoning. The framework supports radiographic decision assistance and does not replace microbiological confirmation, clinical judgment, or definitive diagnoses of tuberculosis.

### 2.11 Parameter selection and robustness considerations

The parameters in the proposed framework were organized into four categories: data-derived parameters, trainable parameters, empirically selected fixed settings, and clinically informed evidence weights. This distinction clarifies which values were calculated from the training data, optimized during model training, or fixed during methodological development. All choices were made within the training and validation workflow. The test partition was not used to revise the final configuration. After adoption, the same settings were used throughout the reported experiments.

The class-balancing coefficient $\left(\alpha_{t}\right)$ in focal loss was calculated inversely from the class frequencies in the training partition. The focusing parameter was fixed at (γ=2.0) to reduce the contribution of well-classified samples while preserving greater influence for difficult and underrepresented observations. This setting supported minority-class learning without destabilizing convergence.

The local–global fusion coefficient was implemented as a sigmoid-constrained trainable scalar. It was initialized at approximately 0.55 and updated jointly with the remaining network parameters. The relative contributions of the RegNetY and ViT representations were therefore learned during optimization rather than assigned manually.

The image-enhancement and lung-mask parameters were defined according to their computational roles. The bilateral-filter values, $\left(\sigma_{s}=1.5\right)$ and $\left(\sigma_{r}=25.0\right)$ , were adopted to suppress high-frequency noise while preserving pulmonary edges. CLAHE used an (8×8) tile grid and a clip limit of 2.0 to improve local contrast without excessive noise amplification.

The lung-mask threshold was defined as the maximum of 0.18 and $\left(0.70\times T_{\text{Otsu}}\right)$ . The lower bound prevented mask collapse in dark radiographs, while the scaling factor retained low-contrast pulmonary regions. Morphological closing and opening restored lung continuity and removed isolated responses.

The attention-processing settings were applied to lesion-level activation maps rather than to the original radiographs. Gaussian smoothing with ( σ=7.0 ) reduced fragmented responses and improved spatial continuity. Soft-fusion coefficients of 0.82 and 0.18 retained the dominant normalized activation while preserving weaker pulmonary responses. Gamma correction with an exponent of 0.75 enhanced low-to-moderate attention values without excessively amplifying strongly activated regions.

The attention threshold of 0.28 and the segmentation threshold of 0.40 served different purposes. The lower value generated sensitive candidate regions, whereas the higher value imposed a stricter constraint before active-contour refinement. The adaptive lesion threshold combined Otsu thresholding with the 62nd percentile of the lesion-score distribution. This percentile was selected through empirical validation on a held-out subset to reduce over-segmentation in heterogeneous lesions.

The Chan–Vese procedure was applied for 35 iterations, followed by morphological cleaning. Once adopted, these settings remained fixed across the test images and were not adjusted for individual cases.

The biomarker weights represented the relative importance of image-derived radiological patterns. Higher weights were assigned to findings with stronger relevance to pulmonary tuberculosis, including cavitary lesions, upper-lobe infiltrates, and fibronodular opacities. Lower weights were assigned to less specific findings.

The biomarker weights were constrained to be non-negative and normalized during aggregation. They therefore supported relative evidence integration rather than absolute diagnostic scoring. These weights were not treated as diagnostic probabilities or independent clinical criteria.

The fuzzy membership parameters transformed continuous support scores into graded evidence. Parameter ( α ) controlled the transition from weak to strong membership, while ( β ) defined the support value associated with a membership of 0.5. The symbolic decision thresholds organized active-TB, obsolete-TB, and non-TB evidence. A separate fusion parameter balanced neural probabilities with symbolic evidence. These values were computational settings rather than universal clinical cut-offs.

Robustness was assessed through the analyses reported in the manuscript. The standalone RegNetY, standalone ViT, and hybrid RegNetY–ViT models were compared under the same evaluation framework. This comparison assessed the contribution of local–global architectural integration rather than the sensitivity of each individual parameter.

Five-fold cross-validation reduced dependence on a single experimental partition and examined consistency across different data splits. Explanation stability was also assessed across the cross-validation partitions and augmentation conditions. Quantitative spatial evaluation used IoU, Dice similarity, sensitivity, precision, and attention coverage to examine agreement among attention responses, refined lesion regions, and the available spatial references.

## 3 Results and interpretability analysis

### 3.1 Classification performance of the RegNetY–ViT hybrid framework

Final classification performance was evaluated on the TBX11K independent patient-level test set, which was excluded from training, cross-validation, hyperparameter optimization, early stopping, and model selection. Class-specific sensitivity, precision, specificity, and *F*1-score were calculated from each confusion matrix using a one-versus-rest formulation. AUC values were obtained from predicted class probabilities, while macro-averages were calculated as the unweighted mean across the five diagnostic classes. Overall accuracy was derived separately from the complete multiclass confusion matrix.

As shown in Table 3, the proposed RegNetY–ViT framework achieved the highest overall accuracy among the evaluated architectures. RegNetY achieved 91.1% accuracy, while ViT achieved 92.3%. The proposed framework increased overall accuracy to 95.8%.

**Table 3:** Class-specific and aggregate classification performance of RegNetY, ViT, and the proposed RegNetY–ViT framework on the independent patient-level TBX11K test set.

Model	Diagnostic class or summary measure	Support	AUC (%)	Sensitivity/Recall (%)	Precision (%)	Specificity (%)	*F*1-score (%)	Overall accuracy (%)
RegNetY	Active and latent TB	11	65.2	63.6	58.3	99.8	60.9	—
	Active TB	185	66.4	64.9	80	98.5	71.6	—
	Healthy	1,000	95.5	95	95.3	96.2	95.1	—
	Latent TB	42	84.6	83.3	74.5	99.5	78.7	—
	Sick_but_non_TB	1,000	94.5	92.7	89.8	91.5	91.2	—
	**Macro-average**	—	**81.2**	**79.9**	**79.6**	**97.1**	**79.5**	—
	**Overall accuracy**	—	—	—	—	—	—	**91.1**
ViT	Active and latent TB	11	57.3	54.5	50	99.7	52.2	—
	Active TB	185	84.5	81.6	82.5	98.4	82.1	—
	Healthy	1,000	95.6	94.9	95.6	96.4	95.2	—
	Latent TB	42	82.3	78.6	73.3	99.5	75.9	—
	Sick_but_non_TB	1,000	94.1	92.7	92.2	93.7	92.5	—
	**Macro-average**	—	**82.8**	**80.5**	**78.7**	**97.6**	**79.6**	—
	**Overall accuracy**	—	—	—	—	—	—	**92.3**
RegNetY–ViT	Active and latent TB	11	65.8	63.6	100	100	77.8	—
	Active TB	185	92.2	89.7	90.7	99.2	90.2	—
	Healthy	1,000	98.6	97.9	97.2	97.7	97.6	—
	Latent TB	42	82.5	78.6	84.6	99.7	81.5	—
	Sick_but_non_TB	1,000	96.7	95.8	95.6	96.4	95.7	—
	**Macro-average**	—	**87.2**	**85.1**	**93.6**	**98.6**	**88.5**	—
	**Overall accuracy**	—	—	—	—	—	—	**95.8**

Bold values indicate aggregate or overall values.

RegNetY achieved a macro-average AUC of 81.2%, sensitivity of 79.9%, precision of 79.6%, specificity of 97.1%, and *F*1-score of 79.5%. ViT produced a macro-average AUC of 82.8%, sensitivity of 80.5%, precision of 78.7%, specificity of 97.6%, and *F*1-score of 79.6%. In comparison, RegNetY–ViT achieved a macro-average AUC of 87.2%, sensitivity of 85.1%, precision of 93.6%, specificity of 98.6%, and *F*1-score of 88.5%.

The consistent improvement across these measures suggests that integrating local convolutional features with global transformer-based contextual representations enhanced discrimination among diagnostic categories. The standalone architectures showed complementary strengths. RegNetY performed strongly for Healthy and Sick_but_non_TB images, but its sensitivity for Active TB was limited to 64.9%. ViT increased Active TB sensitivity to 81.6% and improved the corresponding AUC from 66.4 to 84.5%. This result supports the role of global spatial context modeling in recognizing distributed pulmonary abnormalities.

However, ViT showed weaker performance for Active & Latent TB. It achieved an AUC of 57.3%, sensitivity of 54.5%, precision of 50%, and *F*1-score of 52.2%.

**Figure 2:**
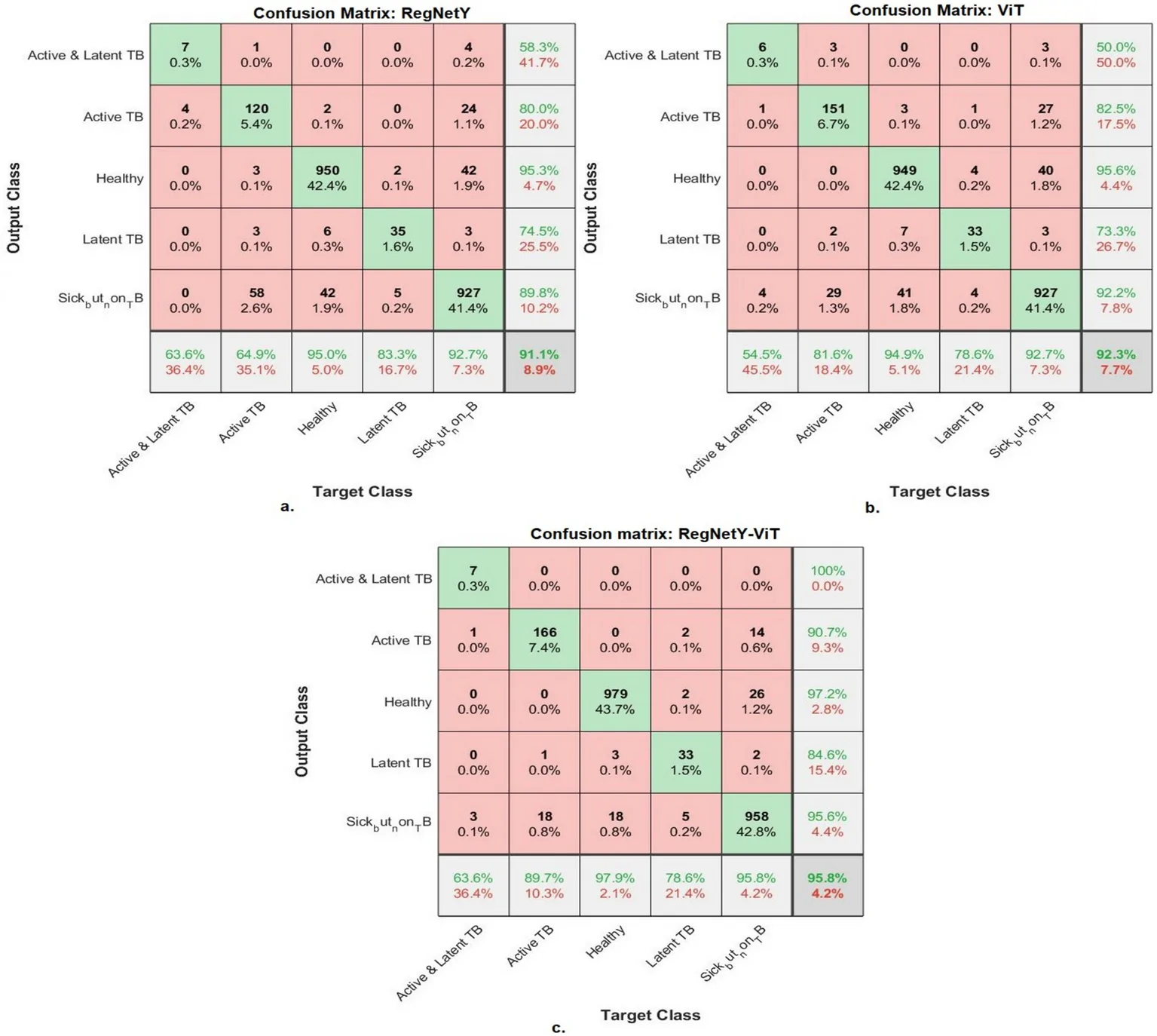
Confusion matrices for evaluating the performance of the proposed chest X-ray classification systems on the TBX11K dataset **(a)** RegNetY model **(b)** ViT model **(c)** proposed RegNetY–ViT hybrid framework.

The largest improvement of the proposed framework was observed for Active TB. It achieved an AUC of 92.2%, sensitivity of 89.7%, precision of 90.7%, specificity of 99.2%, and *F*1-score of 90.2%. According to Figure 2c, 166 of the 185 Active TB images were classified correctly. Eighteen were classified as Sick_but_non_TB, and one was classified as Latent TB. None was classified as Healthy or Active & Latent TB. Most errors therefore occurred between radiographically related abnormal categories rather than between active disease and normal lungs.

The proposed model also maintained strong performance for the majority classes. For Healthy images, it achieved an AUC of 98.6%, sensitivity of 97.9%, precision of 97.2%, specificity of 97.7%, and *F*1-score of 97.6%. It correctly classified 979 of the 1,000 Healthy test images. Eighteen were classified as Sick_but_non_TB, and three were classified as Latent TB.

For Sick_but_non_TB, the framework achieved an AUC of 96.7%, sensitivity of 95.8%, precision of 95.6%, specificity of 96.4%, and *F*1-score of 95.7%. It correctly classified 958 of the 1,000 test images. Most errors involved classification as Healthy or Active TB, reflecting overlap between some non-tuberculous abnormalities and tuberculosis-related radiographic patterns.

Performance remained lower for the rare tuberculosis categories. For Latent TB, the model achieved an AUC of 82.5%, sensitivity of 78.6%, precision of 84.6%, specificity of 99.7%, and *F*1-score of 81.5%. It correctly classified 33 of the 42 test images. For Active & Latent TB, seven of 11 images were classified correctly, three were classified as Sick_but_non_TB, and one was classified as Active TB. Sensitivity was 63.6%, while precision reached 100%.

### 3.2 Multi-lesion localization, visual attention, and biomarker activation analysis

The proposed RegNetY-ViT framework provides interpretable visual and radiological evidence in addition to image-level classification. Having introduced the classification capability in the previous Section, this section investigates the model’s attention map and the relevance of the highlighted lung regions to radiological patterns associated with TB. analyzed multi-lesion Grad-CAM localization, pathology-specific visual attention, and qualitative biomarker activation interpretation on chest radiographs of the TBX11K dataset.

The inputs to this stage were the preprocessed chest X-ray image, the estimated lung-field mask, and the clinician-defined bounding boxes supplied with the dataset. Class-specific Grad-CAM responses generated by the trained classifier were restricted to the corresponding pulmonary analysis domains. These responses served as spatial priors for lesion localization and segmentation. Radiological biomarker responses were subsequently extracted and quantified within the refined lesion regions.

These annotations were not treated as manually delineated pixel-level lesion contours. The final attention responses were generated adaptively from activation distributions within the lung-constrained regions. This distinction is important because rectangular annotations indicate lesion locations but do not reproduce their exact shapes or boundaries. Tuberculosis-related abnormalities may also be irregular, diffuse, or multifocal within the annotated regions.

The hybrid configuration consists of RegNetY local texture extraction and ViT global context modeling. This combination may be helpful as active and inactive pulmonary tuberculosis may occur simultaneously on the same X-ray. The anatomical distribution, opacity characteristics, and structural distortion patterns may vary. Therefore, lesion-specific Grad-CAM responses were generated to enable independent visual analysis of each abnormal pulmonary region while preserving its link to the overall image-level prediction.

Using the RegNetY–ViT framework, attention maps were derived by tracing class-relevant activation responses within each lung lesion domain. The proposed approach differs from the conventional single heatmap, which might combine several regions of abnormal data into a single graphical description. This could minimize the likelihood of being distracted by one abnormality to the detriment of the other. This separation can be beneficial in the management of multifocal tuberculosis, in which active and residual lesions may be present within the same lung zone.

Supportive color-coded heatmaps are provided to assist with visual interpretation by disease. The red attention overlay is used for Active TB regions, and the blue overlay is used for Obsolete Pulmonary Tuberculosis regions. The same color is used for regions with the same disease label; this may help maintain consistency across multiple lesions. For traditional heatmaps, this color assignment could reduce some of the confusion caused by high activation being mistaken for image brightness or artifacts that do not represent activations.

The relationship between neural attention and image-derived radiological evidence was examined after lesion segmentation. Grad-CAM indicated the pulmonary regions that contributed to the predicted class, whereas radiological feature analysis characterized the patterns present within the refined regions. Spatial agreement between these two outputs was interpreted as supportive evidence of consistency, but not as proof that Grad-CAM independently identified a specific radiological biomarker.

**Figure 3:**
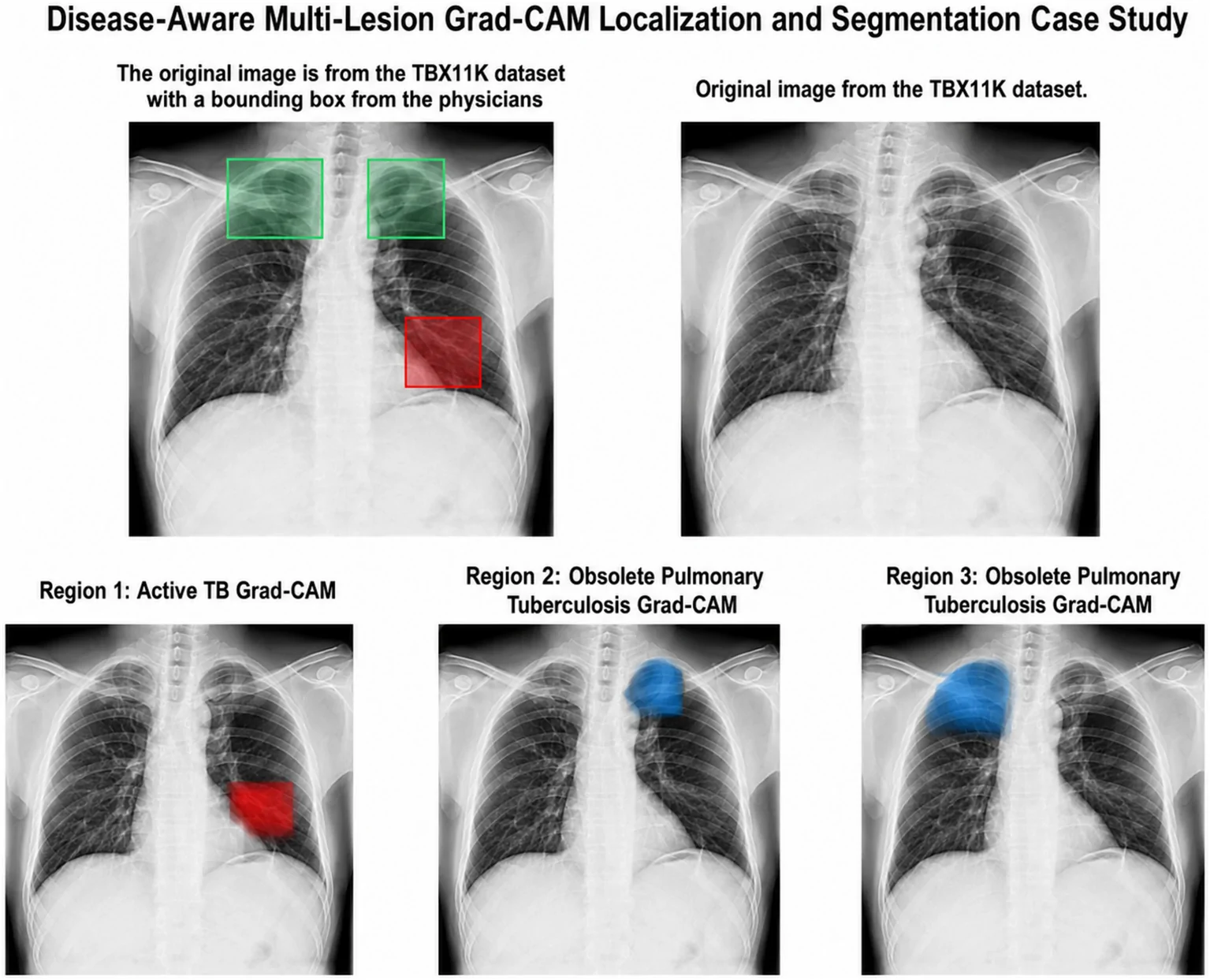
Representative qualitative case study of multi-lesion attention localization using the RegNetY–ViT framework. Active TB (lower left lung, red overlay) and obsolete pulmonary tuberculosis (upper lung zones, blue overlays) are visualized with pathology-specific color-coded Grad-CAM attention maps.

A representative qualitative case study is shown in Figure 3. There are three regions on the radiograph that relate to tuberculosis: one in the lower left lung, consistent with Active TB, and two in the upper lungs, consistent with Obsolete Pulmonary Tuberculosis. In contrast to the spatially diffuse attention pattern over the abnormal lower lung region shown in the red Grad-CAM overlay for the active region, a spatially concentrated attention pattern of the active region was observed. In contrast, the blue shading in the old TB regions appears more widespread, extending throughout the upper lungs. This visual discrepancy suggests that the framework might be capable of maintaining focus on lesions rather than blurring them into a generalized abnormality. This separation enabled analysis of the model output at the lesion level and maintained a visual distinction between active and obsolete TB findings within the same image.

The highlight of this section is the combination of lesion-wise Grad-CAM localization, pathology-specific visualization, and biomarker-based interpretation. Although conventional Grad-CAM can identify important regions in an image, it fails to explain their radiological significance, particularly when multiple abnormalities are present. This limitation is addressed in the proposed approach by decoupling lesion attention and associating each response with visual biomarkers of tuberculosis. This offers a possible avenue of multi-level interpretability: image-level prediction, lesion-level localization, disease-specific visual attention, and biomarker-assisted reasoning.

### 3.3 Quantitative lesion-localization and segmentation-agreement evaluation

Fifty radiographs with available clinician-annotated lesion regions were selected from the fixed independent TBX11K test set using a predefined sampling procedure. All selected radiographs were excluded from model development. The same localization and segmentation pipeline was applied independently to each image, and all thresholds and post-processing parameters were fixed before the expanded evaluation.

The evaluation was performed at the lesion level. When multiple lesions appeared on a single radiograph, each region was processed and evaluated separately. Combined overlap measures were also calculated for multi-lesion radiographs as a secondary image-level analysis.

TBX11K clinician-annotated bounding boxes were used only to define lesion search regions. A reference lesion mask was derived within each bounding-box region by applying pulmonary-region restriction, adaptive contrast enhancement, texture and edge analysis, morphological processing, and active-contour optimization. The bounding-box area itself was not treated as a segmentation mask.

The attention-based prediction mask was generated independently using model-derived activation, thresholding, morphological regularization, and active-contour refinement. The ROI-derived reference lesion mask was used only after prediction to calculate spatial-agreement metrics. It was not used to restrict or refine the predicted mask.

For each lesion region, IoU, Dice coefficient, sensitivity, and precision were calculated as Equations 64–67:


$$\mathrm{IoU}=\frac{|P\cap R|}{|P\cup R|}$$ (64)

$$\text{Dice}=\frac{2|P\cap R|}{|P|+|R|}$$ (65)

$$\text{Sensitivity}=\frac{|P\cap R|}{|R|}$$ (66)

$$\text{Precision}=\frac{|P\cap R|}{|P|}$$ (67)

where *P* denotes the predicted lesion mask and *R* denotes the ROI-derived reference lesion mask. Results were summarized using the mean, standard deviation, median, minimum, and maximum across all evaluated lesion regions.

To determine whether the spatial-agreement results extended beyond the representative example, an expanded quantitative evaluation was conducted on 50 radiographs from the independent TBX11K test set. IoU, Dice coefficient, lesion sensitivity, and lesion precision were calculated for each lesion.

As shown in Table 4, the proposed procedure achieved a mean IoU of 0.82 ± 0.08 and a mean Dice coefficient of 0.89 ± 0.05. The corresponding median values were 0.84 and 0.90. Their proximity to the mean values suggests that the distributions were not markedly skewed by a small number of extreme cases.

**Table 4:** Lesion-level spatial agreement across 50 independent TBX11K test radiographs.

Metric	Mean ± SD	Median	Minimum	Maximum
IoU	0.82 ± 0.08	0.84	0.63	0.93
Dice coefficient	0.89 ± 0.05	0.9	0.77	0.96
Lesion sensitivity (%)	90.8 ± 5.4	91.6	79.5	98.1
Lesion precision (%)	91.5 ± 4.8	92.1	81.4	98.4

Mean lesion sensitivity reached 90.8 ± 5.4%, while lesion precision was 91.5 ± 4.8%. The similarity between these values indicates balanced coverage of the ROI-derived reference regions and control of predicted pixels outside those regions. The results, therefore, did not reflect the high lesion coverage achieved at the expense of substantial spatial overprediction.

IoU ranged from 0.63 to 0.93, and Dice values ranged from 0.77 to 0.96. These ranges demonstrate measurable case-level variability across the evaluated lesion regions. The standard deviations of 0.08 for IoU and 0.05 for Dice also indicate moderate variation rather than uniformly ideal performance.

### 3.4 Lung-constrained lesion segmentation and quantitative agreement analysis

Segmentation converts attention-based localization into an anatomically defined lesion region. The present analysis evaluated whether these responses could be translated into candidate lesion regions showing regional overlap with coarse bounding-box-derived references. Therefore, quantitative assessment relies on spatial agreement between reference and predicted lesion masks rather than visual attention patterns alone.

**Figure 4:**
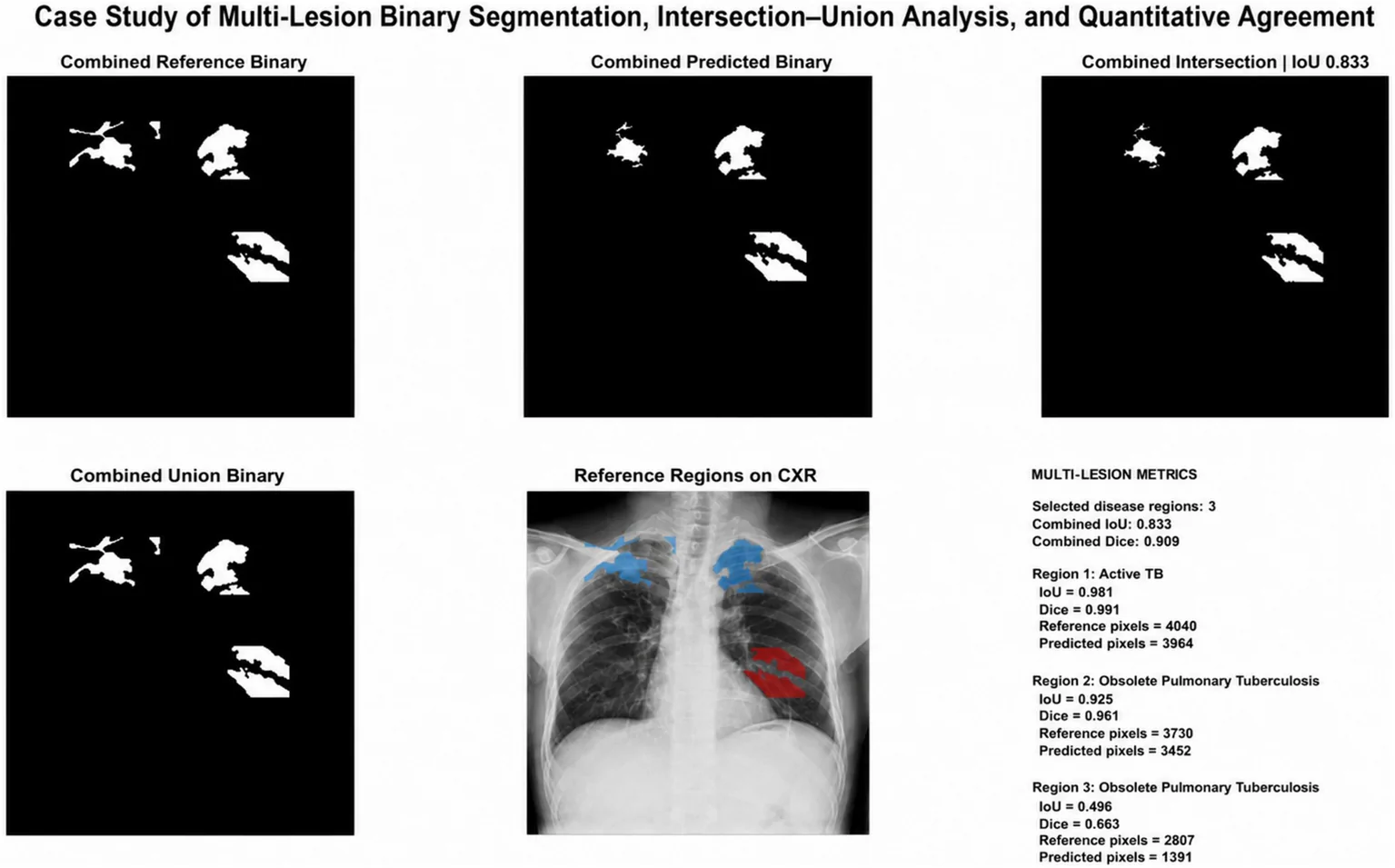
Multi-lesion lung-constrained segmentation and quantitative spatial-agreement analysis showing clinician-defined bounding-box-derived reference masks, predicted lesion masks, intersection and union regions, and lesion-wise overlap measures for one active TB lesion and two obsolete pulmonary tuberculosis lesions.

Figure 4 presents a representative multi-lesion case containing one Active TB lesion and two Obsolete Pulmonary Tuberculosis lesions. Each lesion was processed independently within a lung-constrained domain. The predicted lesion masks were then merged for image-level evaluation.

The reference annotations consisted of clinician-defined bounding boxes supplied with the dataset. These annotations indicated the approximate locations of the pulmonary lesions. The bounding boxes guided lesion extraction within the pulmonary ROIs and were represented as binary rectangular reference masks for quantitative evaluation.

The predicted masks were generated from lung-constrained Grad-CAM activations using adaptive thresholding, Chan–Vese active-contour refinement, and morphological regularization. Restricting the segmentation process to pulmonary tissue reduced false-positive extensions into the ribs, mediastinal structures, and surrounding soft tissues.

The predicted lesion masks showed different levels of spatial agreement with the bounding-box-derived reference masks. Intersection maps identified areas shared by the predicted masks and the rectangular reference regions. Union maps represent the total area covered by both spatial representations.

Because the reference masks were derived from rectangular bounding boxes, the reported IoU and Dice values mainly reflect localization and regional overlap. They do not measure precise agreement with manually delineated lesion boundaries.

Region 1, corresponding to Active TB, achieved an IoU of 0.981 and a Dice coefficient of 0.991. The reference region contained 4,040 pixels, whereas the predicted mask contained 3,964 pixels. These results indicate strong spatial agreement between the predicted region and the clinician-annotated lesion location.

However, the reference region was derived from a rectangular bounding box rather than an exact lesion contour. The result therefore reflects high localization consistency and regional overlap. It should not be interpreted as exact pixel-level boundary agreement.

Region 2, corresponding to Obsolete Pulmonary Tuberculosis, also demonstrated strong spatial agreement. The region achieved an IoU of 0.925 and a Dice coefficient of 0.961. The reference region contained 3,730 pixels, whereas the predicted mask contained 3,452 pixels.

Although the predicted region was slightly smaller than the rectangular reference region, the overlap remained substantial. This finding indicates that the model localized the principal abnormality within the clinician-annotated pulmonary area. It does not establish exact agreement with a manually delineated lesion contour.

A different pattern was observed in Region 3. The system generated a warning indicating weak lung-mask support within the ROI. The bounding-box-derived reference mask contained 2,807 pixels, whereas the predicted lesion mask contained 1,391 pixels. The resulting IoU and Dice coefficient were 0.496 and 0.663, respectively.

The lower agreement indicates that the predicted region covered only part of the annotated pulmonary location. This difference may reflect weak lung-boundary support, low lesion contrast, or diffuse chronic abnormalities. It may also result from the rectangular reference region containing surrounding non-lesion tissue. The lower overlap should therefore not be attributed solely to segmentation failure.

At the combined image level, the predicted regions achieved an IoU of 0.833 and a Dice coefficient of 0.909. These values summarize spatial agreement across three pulmonary regions with different anatomical locations and radiographic characteristics.

Despite the lower overlap in Region 3, the combined results indicate strong overall consistency in localization. However, these values were calculated using coarse rectangular references. They should not be interpreted as agreement with exact expert-delineated lesion boundaries.

Figure 4 presents a representative multi-lesion example illustrating the localization workflow and the separate processing of three lesion regions. The figure is provided only as a visual explanation. The aggregate quantitative results are reported independently in Table 4.

Table 5 presents the lesion-level spatial agreement for the representative multi-lesion case. Region 1, corresponding to Active TB, achieved an IoU of 0.981 and a Dice coefficient of 0.991. These values indicate strong overlap with the clinician-defined bounding-box-derived reference region. Region 2, corresponding to Obsolete Pulmonary Tuberculosis, also maintained strong spatial agreement, with an IoU of 0.925 and a Dice coefficient of 0.961.

**Table 5:** Lesion-level and combined segmentation agreement in the representative multi-lesion case.

Region	Radiological interpretation	Reference pixels	Predicted pixels	IoU	Dice coefficient	Main observation
Region 1	Active TB	4,040	3,964	0.981	0.991	Strong spatial overlap with the bounding-box-derived reference region
Region 2	Obsolete pulmonary tuberculosis	3,730	3,452	0.925	0.961	High spatial agreement despite heterogeneous fibrotic morphology
Region 3	Obsolete pulmonary tuberculosis	2,807	1,391	0.496	0.663	Partial regional overlap associated with weak lung-mask support and a coarse rectangular reference
Combined image	Active and obsolete abnormalities	—	—	0.833	0.909	High overall localization and regional-overlap consistency across three spatially distinct lesions

Region 3 showed lower spatial agreement, with an IoU of 0.496 and a Dice coefficient of 0.663. This reduction may be associated with weak lung-mask support, diffuse chronic abnormalities, and the rectangular form of the reference annotation. The combined image-level IoU of 0.833 and Dice coefficient of 0.909 indicate high overall localization and regional-overlap consistency across the three lesions. These values should be interpreted relative to bounding-box-derived spatial references rather than to exact expert-delineated lesion contours.

### 3.5 Neuro-symbolic clinical inference results

The final step in the proposed RegNetY–ViT framework is translating quantitative imaging evidence into an explainable clinical decision-support outcome via a neuro-symbolic fuzzy inference mechanism. This framework combines lesion-specific attention responses, lung-constrained segmentation outputs, radiological biomarker activations, and disease-specific inference rules to produce interpretable diagnostic evidence that is not solely based on the classification probabilities. Grad-CAM attention maps, fine-tuned abnormality masks, normalized biomarker activation maps, and weighted biomarker support scores from the previous layer were fed into this layer. The quantitative results are then converted to symbolic clinical evidence and consolidated by the neuro-symbolic reasoning process to obtain the final image-level inference.

**Figure 5:**
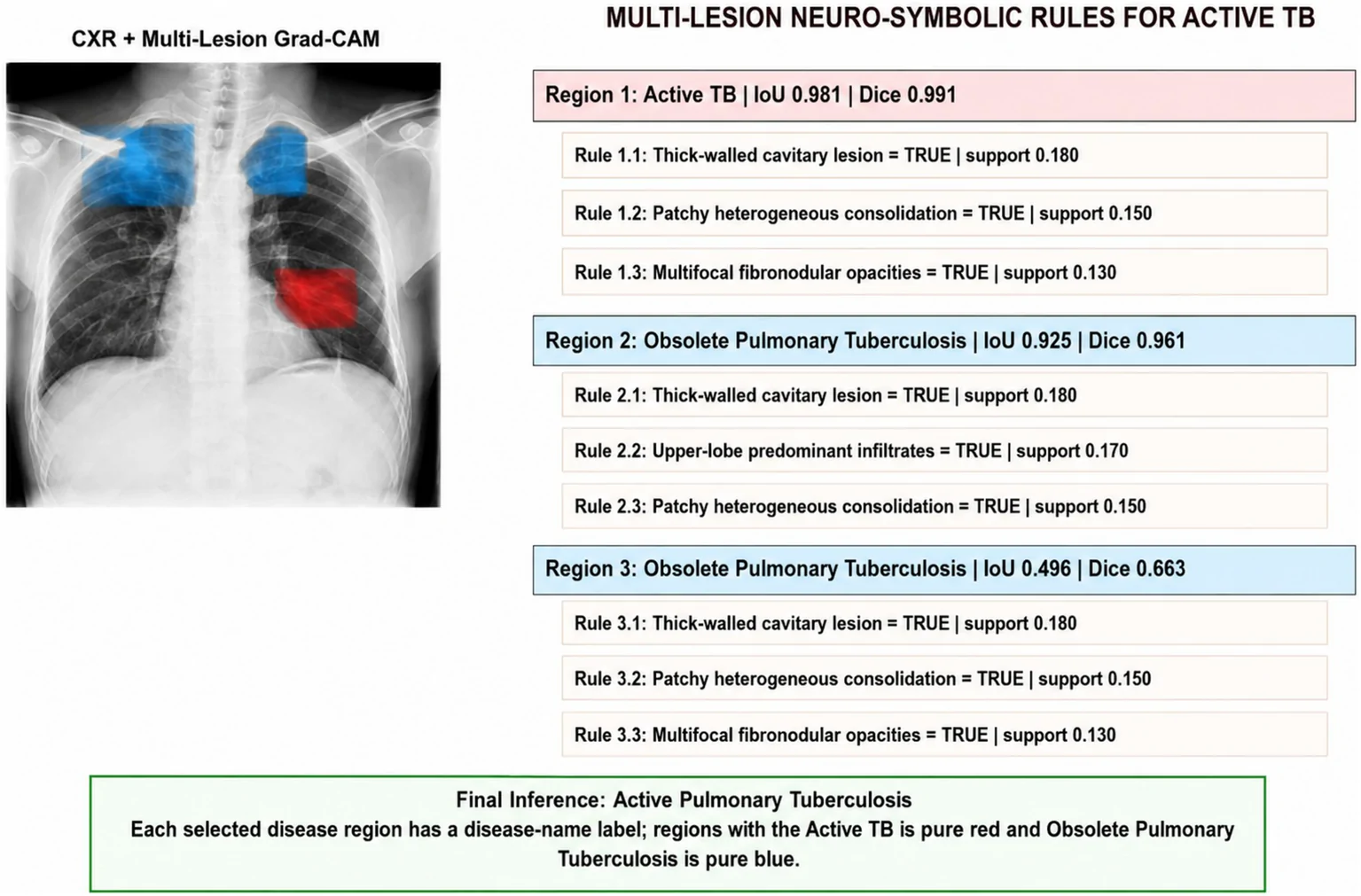
Neuro-symbolic clinical inference for a multi-lesion tuberculosis case. Red overlay: Active TB. Blue overlays: Obsolete TB. Right panel shows activated rules, support scores, IoU/Dice, and final model-derived inference.

Figure 5 shows a representative multi-lesion case study with three pulmonary abnormalities. The lower left lung area was diagnosed with Active Tuberculosis, whereas the two upper lung regions were diagnosed with Obsolete Pulmonary Tuberculosis. The visualization strategy suggested by the disease-aware approach overlays a red layer on active tuberculosis and a blue layer on healed pulmonary tuberculosis, allowing both active and healed tuberculosis to be visualized on the same chest radiograph. This is of clinical importance because many patients with pulmonary tuberculosis also have active infectious foci and residual post-tuberculosis abnormalities.

The first step in the inference process was to calculate the support scores for the biomarkers in each abnormal region. With support values of 0.180, 0.150, and 0.130, respectively, the most frequently associated activated biomarkers in the active tuberculosis region were thick-walled cavitary lesions, patchy heterogeneous consolidation, and multifocal fibronodular opacities. These are well-known radiographic markers of TB activity, and together they provide the strongest evidence of active disease. The predicted lesion region showed strong spatial agreement with the bounding-box-derived reference region. It achieved an IoU of 0.981 and a Dice coefficient of 0.991. These measures support consistent localization within the clinician-annotated pulmonary area. However, they do not establish exact lesion-boundary accuracy because the reference region was derived from a rectangular bounding box. The biomarker support scores provide structured image-derived evidence for the subsequent symbolic reasoning stage.

In the upper pulmonary regions, the created framework inferred Obsolete Pulmonary Tuberculosis. In Region 2, the IoU was 0.925, and the Dice coefficient was 0.961, with strong activation of thick-walled cavitary lesions, upper lobe predominant infiltrates, and patchy heterogeneous consolidation. The activation of thick-walled cavitary lesions and patchy heterogeneous consolidation and multifocal fibronodular opacities were observed in Region 3 with an IoU of 0.496 and a Dice coefficient of 0.663. The system indicated a weak lung mask within the third region during processing, suggesting greater uncertainty in the pulmonary boundary estimation. The lower overlap in this region may be associated with reduced lung-mask support and the diffuse appearance of chronic abnormalities. It may also reflect the rectangular shape of the reference annotation, which can include surrounding pulmonary tissue. Therefore, the lower overlap should not be attributed exclusively to failure of the segmentation or inference framework.

The proposed framework performs lesion-specific reasoning before reaching an image-level conclusion, whereas conventional deep learning classifiers can only provide an image-level label. The entire inference is drawn from evidence aggregated across all identified abnormalities; each analyzed separately and assigned a disease-specific evidence profile. This allows for individuality in findings across distinct spatial locations while enabling a common interpretation of multifocal disease patterns within the same region. The regularity of the localization, segmentation, and evidence pathway from biomarker quantification to symbolic reasoning is explainable, thereby highlighting the consistency of this pathway across these steps, from image to clinically relevant radiological evidence, culminating in a final decision. It is also worth noting that the accuracy of neuro-symbolic reasoning still relies on the accuracy of the outputs from the extraction and localization of biomarkers in the previous stages.

The final inference of the detected radiographic abnormality that was used to draw the conclusion of the analysis was Active Pulmonary Tuberculosis, based on the aggregated evidence extracted from all detected abnormalities. This is a clinically conceivable result as a result of the fact that even if there is a dominant active tuberculosis focus, the examination will still be considered active tuberculosis. In general, the results show that the proposed neuro-symbolic framework is capable of explaining diagnostic reasoning in a transparent manner that is lesion-specific while maintaining interpretability for explainable clinical decision-support systems.

### 3.6 Evaluation of the RegNetY–ViT architecture on an independent dataset

For external validation, we used the entire Dataset of Tuberculosis Chest X-ray Images, an independent, publicly available dataset collected from a local hospital in Pakistan. The dataset comprises 2,494 chest X-ray images of patients with tuberculosis and 514 normal chest X-ray images. All images were preprocessed and resized to a uniform dimension, ensuring consistency for model evaluation. The complete dataset was used for external testing to comprehensively assess the generalizability, robustness, and interpretability of the proposed RegNetY–ViT model beyond the TBX11K dataset. Its two-class structure provides a suitable benchmark for tuberculosis-versus-normal classification, while its independent clinical source offers a reliable evaluation of the model’s performance on unseen data. Furthermore, the dataset is publicly available under the CC BY 4.0 license, supporting transparent and reproducible research.

External validation was performed on the complete independent dataset to assess the generalizability of the proposed RegNetY–ViT model beyond TBX11 K.

Table 6 presents the classification performance of the proposed RegNetY–ViT architecture on the independent tuberculosis chest X-ray dataset. The model achieved an overall accuracy of 92.5%, demonstrating strong performance on images that were not included in the original TBX11K dataset. The average AUC was 85.65%, while the average sensitivity, precision, and specificity were 85.75, 87.4, and 85.95%, respectively. These results indicate that the model maintained a high overall classification ability during external validation.

**Table 6:** Classification performance of the RegNetY–ViT architecture on the independent dataset.

Models	Classes	AUC %	Sensitivity %	Accuracy %	Precision %	Specificity %
RegNetY-ViT	Normal chest X-rays	76.7	75.1	75.1	79.9	96.5
	TB chest X-rays	94.6	96.1	96.1	94.9	75.4
	**Average ratio**	**85.65**	**85.75**	**92.5**	**87.4**	**85.95**

Bold values indicate aggregate or overall values.

For the normal chest X-ray class, the model obtained an AUC of 76.7%, sensitivity of 75.1%, accuracy of 75.1%, and precision of 79.9%. Its specificity for this class reached 96.5%. According to the confusion matrix, 386 of 514 normal images were classified correctly. However, 128 normal images were incorrectly classified as tuberculosis. Thus, the model correctly identified approximately three-quarters of the normal cases, while 24.9% of them were assigned to the TB class.

The model showed substantially stronger performance for tuberculosis detection. It achieved an AUC of 94.6%, sensitivity of 96.1%, accuracy of 96.1%, and precision of 94.9% for the TB class. The corresponding specificity was 75.4%. Of the 2,494 tuberculosis images, 2,397 were correctly classified as TB, whereas only 97 images were incorrectly classified as normal. Therefore, the model detected approximately 96.1% of the tuberculosis cases, with a misclassification rate of only 3.9%.

The external validation results show that RegNetY–ViT generalizes effectively to an independent clinical dataset. Its main strength is the reliable detection of tuberculosis, as reflected by the high TB sensitivity, accuracy, precision, and AUC values. Nevertheless, the lower performance for the normal class indicates a tendency to classify some normal images as tuberculosis. This behavior increases false-positive predictions but reduces the risk of missing actual TB cases, which is particularly important in tuberculosis screening applications.

## 4 Discussion and comparative interpretation

A high predictive performance is not the only challenge in TB image analysis. High accuracy is insufficient for clinical use if the decision-making process is opaque. The major question is not just whether the model predicts correctly, but whether it predicts based on clinically relevant radiological reasoning. Radiographs of the chest frequently show overlapping features, such as active lesions, fibrotic remnants, and multiple disease phases. Such complexity demands that interpretability be considered a fundamental design goal, not an afterthought. The proposed RegNetY–ViT meets this requirement by providing an integrated framework encompassing representation learning, lesion localization, lesion segmentation for validation, biomarker interpretation, and neuro-symbolic reasoning.

However, interpretability in this context is not limited to a single module; it is built up over several steps of analysis. RegNetY and ViT were pivotal in this process. RegNetY is highly sensitive to local textures and can detect fine-grained radiological patterns related to pulmonary abnormalities. This is complemented by ViT, which recognizes the spatial dependency between thoracic structures at the global level. The dual representation reduces feature misinterpretation and helps separate individual pathological areas. The framework does not generate overlapping or diffuse responses but rather lesion-specific responses that mimic the diversity of TB.

The first layer of interpretability is multi-lesion localization, which separates independent abnormal regions in identical radiographs. This separation is clinically significant because tuberculosis rarely presents as a single continuous lesion. Rather, active and residual abnormalities can coexist. The framework does not consider attention maps to be final explanations but rather spatial indications for further validation with anatomical and clinical consistency.

The second layer of interpretability comes from biomarker activation analysis, in which visual patterns are linked to known radiological concepts. Cavitary appearance, upper lobe infiltrates, and fibronodular changes are not considered abstract activations but rather clinically significant findings. This mapping allows for moving from feature-based learning to evidence-based interpretation. In doing so, the model’s representations shift toward clinically recognizable patterns rather than statistical correlations.

The lung-constrained segmentation stage imbues interpretability with a quantitative aspect. Segmentation not only refers to visual attention but also describes the anatomic boundaries of suspected abnormalities in the pulmonary field. The framework enables analysis of lung regions, thereby minimizing the influence of non-pulmonary structures and artifacts. Evaluating the model’s focus on clinically relevant areas involves assessing spatial agreement between the regions of interest and segmented lung abnormalities, which is an objective measure. This step enhances interpretability by introducing spatial consistency as a measurable indicator of visual plausibility.

Quantitative validation is important for this process. While a visual explanation might seem convincing, it may also be a spurious correlation unless objectively evaluated. Spatial agreement metrics provide a means to distinguish clinically relevant attention from visually plausible yet diagnostically irrelevant patterns. This distinction is important to ensure that interpretability is based on anatomical actuality rather than subjective visualization.

The last interpretability stage is represented by neuro-symbolic inference. The framework is based on biomarker evidence rather than probability-based outputs. Each radiological feature will contribute based on its assigned weighted fuzzy membership value. These contributions are combined with rule-based logic, and a final decision is made. This process is more representative of clinical reasoning than traditional classification models, which lack access to intermediate reasoning. This is not only a predicted label but also a structured representation of the evidence that supports it.

Initial segmentation studies focused mainly on lesion localization. Ou et al. (7) enhanced the ability to delineate the lesions by applying ensemble U-Net variants, while Sharma et al. (8) added three U-Net variants, namely for lung segmentation, classification, and visualization using Grad-CAM. These studies improved spatial localization but provided little insight into how localized abnormalities informed diagnostic reasoning. This indicates that there was no strong link between segmentation performance and clinical interpretability.

Another research focus is predictive optimization. Strong classification performance was obtained using hybrid CNN architectures, fusion strategies, and specialized optimization strategies. Ali et al. (9), Kumar et al. (10), and Ragavan et al. (11) have shown that deep learning models can identify patterns in TB. However, these methods provide little insight into radioactive data to support their forecasts. High performance led to more accurate diagnoses, but the reasoning process remained unavailable. This is a limitation that must not be overlooked when considering the clinical trust required to make predictions that can be interpreted as decisions.

To address some of the drawbacks of traditional CNN systems, transformer and multimodal architectures have been proposed. Research by Lu et al. (12), Ammar et al. (13), Kumar et al. (14), and Kotei et al. (15) demonstrated the importance of contextual modeling and learning with long-range dependencies. These strategies provided higher-quality representation and diagnostic discrimination. However, most interpretations are still performed using *post hoc* visualization methods. For the attention maps, suspicious regions were identified, but little could be deduced about whether these regions were clinically significant. Only a limited quantitative assessment of attention behavior is available.

To clarify the methodological distinction between the proposed framework and previous tuberculosis imaging studies, Table 7 summarizes representative segmentation, convolutional, transformer, multimodal, and explainable-AI approaches. The comparison considers the imaging task, architecture, reported performance, interpretability method, quantitative explanation validation, and clinical reasoning capability. Previous studies have generally concentrated on either predictive performance, lesion delineation, or visual explanation. In contrast, the proposed framework integrates local–global representation learning, lesion-specific attention, lung-constrained segmentation, quantitative spatial validation, biomarker-response analysis, and fuzzy neuro-symbolic inference within one traceable computational pathway.

**Table 7:** Comparison of representative automated tuberculosis imaging approaches and the proposed framework.

Study	Primary task	Main architecture or method	Reported result	Interpretability component	Quantitative explanation validation	Structured clinical reasoning
Ou et al. (7)	TB lesion segmentation	Ensemble of U-Net variants, including Attention U-Net++ and PSP Attention U-Net	MIoU = 0.70; *F*1-score = 0.81	Attention-based segmentation	Segmentation overlap metrics	No
Sharma et al. (8)	Lung segmentation and TB classification	U-Net, Xception, and Grad-CAM	Reported classification and localization performance	Grad-CAM visualization	Limited to the segmentation and classification outputs	No
Ali et al. (9)	TB classification	EfficientNetB0–ResNet50 feature fusion	Accuracy above 99%	Not incorporated	No	No
Kumar et al. (10)	TB classification	CNN with orthogonal SoftMax layer	Improved discrimination on limited data	Not incorporated	No	No
Ragavan et al. (11)	TB classification	Hybrid sea lion optimization and political optimizer	Strong classification performance	Not incorporated	No	No
Lu et al. (12)	TB classification	CTBViT with patch reduction blocks	Improved token efficiency and classification	Transformer attention	No objective lesion-level validation reported	No
Ammar et al. (13)	TB classification	Vision transformer–EfficientNet fusion	Strong diagnostic performance	Model attention visualization	No spatial agreement analysis reported	No
Kumar et al. (14)	Multimodal TB recognition	Cross-attention transformer using imaging and clinical information	Improved multimodal discrimination	Cross-attention representation	No lesion-mask agreement analysis reported	Limited to multimodal prediction
Kotei et al. (15)	TB classification on TBX11K	Data-efficient image transformer and ResNet-16	High classification accuracy	Transformer attention	No integrated biomarker and rule validation reported	No
Rahman et al. (16)	Explainable TB classification	Deep classifier with Score-CAM	Classification with visual explanation	Score-CAM	Primarily qualitative	No
Maheswari et al. (17)	Explainable TB classification	Deep classifier with CAM or related visualization	Classification with visual explanation	CAM-based localization	Primarily qualitative	No
Nafisah et al. (18)	Explainable TB classification	Deep learning with LIME and visual explanation	Classification with local explanation	LIME	Primarily qualitative	No
Proposed RegNetY–ViT framework	Five-class TB classification, localization, segmentation, and clinical evidence generation	Lightweight RegNetY-inspired CNN, six-block ViT, learnable fusion, multi-lesion Grad-CAM, lung-constrained segmentation, and fuzzy inference	Overall accuracy = 95.8%; macro-average sensitivity = 85.1%; macro-average specificity = 98.6%; macro-average AUC = 87.2%	Multi-lesion Grad-CAM and biomarker-response maps	IoU, Dice, sensitivity, precision, attention coverage, and rule agreement	Yes; fuzzy rules integrate neural probabilities and radiological evidence

The proposed RegNetY–ViT framework addresses these shortcomings by combining multi-lesion localization, biomarker-guided interpretation, lung-constrained segmentation accompanied by quantitative agreement analysis, and neuro-symbolic clinical inference. Interpretability is not a step in the visualization process but rather an integral step in the diagnostic process. This integration creates a traceable route from image representation to anatomical evidence and radiological biomarkers, and ultimately to diagnostic reasoning.

The interpretability evaluation used the clinician-defined bounding boxes supplied with the TBX11K dataset. These annotations provided clinically informed spatial references for assessing Grad-CAM responses and predicted lesion regions. However, they represented coarse rectangular lesion locations rather than expert-delineated pixel-level contours.

The biomarker and neuro-symbolic components were evaluated through their computational consistency with the localized image responses. The extracted biomarkers represented image-derived radiological patterns based on intensity, texture, opacity, edge, anatomical location, and attention information. The activated rules provided a traceable explanation of how these responses contributed to the model output. These components were not treated as independently confirmed clinical diagnoses.

The present study did not include an independent blinded review of the interpretability outputs by multiple radiologists. Therefore, formal inter-observer agreement could not be calculated. A structured multi-observer assessment was not feasible during the study period because a panel of radiologists with the required thoracic imaging expertise was unavailable.

Accordingly, the reported findings demonstrate computational agreement with clinician-defined dataset annotations. They also demonstrate internal consistency across the interpretability pipeline. However, they do not constitute complete independent clinical validation.

The class imbalance in TBX11K represents an important limitation. The Healthy and Sick_but_non_TB categories each contain 5,000 images, whereas Latent TB and Active & Latent TB contain only 212 and 54 images, respectively. Although minority-class augmentation, focal loss, and five-fold cross-validation were applied, performance estimates for the rare categories remain less stable because of their limited sample sizes. Dataset-related bias also limits generalizability. Differences in patient characteristics, disease prevalence, imaging equipment, acquisition protocols, image processing, annotation practices, and referral patterns can affect model performance.

Future work will include blinded evaluation by multiple thoracic radiologists and expert delineation of pixel-level lesion contours. It will also compare expert annotations with model-generated regions. Clinical verification of the extracted biomarkers and inter-observer agreement analysis of the neuro-symbolic explanations will also be required.

## 5 Conclusion

The interpretation results indicate that the proposed RegNetY–ViT hybrid system is not merely a classifier that outputs a label. It also provides a traceable sequence of attention responses, candidate lesion regions, feature-response scores, and symbolic evidence. This is important when assessing chest radiographs, as TB does not produce a clean, textbook-shaped pattern. It may present as a cavity, a cloudy opacity, a fibrotic scar, or as several old and active signs on the same film.

In contrast to flattening the results into a single heatmap, Multi-lesion Grad-CAM attention localization was able to distinguish these results. This was demonstrated in the representative TBX11K case, which contained one active-TB region in the lower lung and two obsolete-pattern regions in the upper lungs. The attention maps highlighted separate pulmonary regions contributing to the model output rather than producing a single diffuse whole-image visualization.

Lung-constrained lesion segmentation provided an additional quantitative test of regional localization consistency. The framework constrained the analysis to the lungs, making it difficult to be distracted by activation from the ribs, borders, or neighboring structures. The two lesions with higher IoU and Dice values showed good agreement between the predicted and reference masks, whereas the third lesion proved challenging due to uncertain lung boundaries, which reduced the estimated abnormal volume.

The interpretation was endowed with a clinical lexicon through radiological biomarker extraction. These were no longer features that disguised cavitary evidence, upper-lobe infiltrates, consolidation, fibronodular opacity, or fibrotic distortion, but rather were measurable supports. These supports were then combined in a neuro-symbolic fuzzy inference model to form an uncertainty-aware decision. This is not a diagnosis system in the sense that it does not make a final diagnosis. It provides a clear, structured argument that can be examined, challenged, and carefully considered by clinicians.

## Data Availability

Publicly available datasets were analyzed in this study. This data can be found at: the TBX11K dataset used in this study for tuberculosis detection and lesion localization in chest X-ray images was obtained from a publicly available online repository and can be accessed via the following link: https://datasetninja.com/tbx-11k#citation. Importantly, it includes physician-annotated bounding-box (BBOX) labels that highlight clinically relevant regions of abnormality within the lung fields, enabling lesion localization in addition to classification tasks.
